# A comprehensive benchmark of sequence-based subcellular localization predictors for human proteins

**DOI:** 10.1038/s41592-026-03142-6

**Published:** 2026-07-06

**Authors:** Zoe Wefers, Ankit Gupta, Noorsher Ahmed, Xikun Zhang, Emma Lundberg

**Affiliations:** 1 Computer Science Department, Stanford University, Stanford, CA USA; 2 Bioengineering Department, Stanford University, Stanford, CA USA; 3 Science for Life Laboratory, School of Engineering Sciences in Chemistry, Biotechnology and Health, KTH Royal Institute of Technology, Stockholm, Sweden

**Keywords:** Protein function predictions, Machine learning, Proteins, Systems biology

## Abstract

Computational sequence-based predictors of protein localization have the potential to accelerate the discovery of protein functions and interactions, thereby advancing our understanding of human biology and disease. While many methods have been proposed, evaluations remain limited by small test sets, coarse-grained cellular compartment labels and single-label classification, despite the fact that nearly half of human proteins localize to multiple compartments. Here we integrate annotations from major protein databases to construct a highly validated, twofold larger benchmark test set of 3,814 human proteins. Using this dataset, we systematically evaluate existing sequence-based predictors and compare combinations of protein language models and aggregation strategies. We find that current models underperform on fine-grained compartments, multilocalizing proteins and pathogenic variants known to mislocalize. Our results reveal fundamental limitations of existing approaches and underscore the need for improved models, standardized benchmark datasets and more rigorous evaluation in subcellular localization prediction.

## Abstract (web summary)

This Registered Report presents the results of a study to evaluate the performance of sequence-based protein localization predictors on a curated test dataset of 3,814 human proteins.

## Main

Cellular compartmentalization is essential to the complex processes that sustain eukaryotic life. Just as the human body is composed of functional units such as tissues and organs, cells also organize their internal environments through membranes, membraneless condensates and organelles. Each compartment maintains local biochemical conditions optimized for specific functions. For example, energy metabolism occurs in mitochondria, degradation in lysosomes, ribosome biosynthesis in nucleoli, and DNA regulation in the nucleus. Proteins both shape these environments and perform most cellular functions. Thus, determining the subcellular localization of human proteins offers critical insight into their roles and the molecular mechanisms underlying health and disease^1^. Accurate computational predictors of localization could help identify sequence motifs that trigger protein trafficking, functionally annotate uncharacterized proteins, and explain how protein mislocalization plays a role in a number of human diseases^2–4^.

Numerous methods have been developed to predict protein localization^5–10^, and most modern approaches rely on amino-acid sequence data due to its abundance and the success of deep learning for modeling biological sequences^11^. DeepLoc was the first deep-learning model for this task. It used convolutional and recurrent neural networks to predict single-label localizations from the sequence alone^7^. Other models followed, including MULocDeep, which uses a Bidirectional Long Short-Term Memory (BiLSTM)^12^ with attention to predict both subcellular and suborganellar locations^10^. Both models rely on multiple-sequence alignments to extract per-residue features, but a critical development was the adoption of protein language models (PLMs), which are trained on millions of unannotated sequences to learn rich and contextual per-residue protein representations. Recent protein localization models, like DeepLoc2 (ref. ^9^) and LAProtT5 (ref. ^8^), leverage PLMs, specifically ProtT5 (ref. ^13^) and ESM1 (ref. ^14^) and use attention mechanisms to aggregate features across residues.

Despite these advances, each model is limited in at least one key way. Several models only support single-label classification^7,8^, overlooking the fact that nearly half of human proteins localize to multiple compartments, where they often performing distinct functions^15–17^. Others, following the example set by DeepLoc, restrict predictions to ten coarse-grained compartments^7–9^, ignoring finer cellular structures and the full diversity of subcellular architecture. Further, some methods are evaluated primarily on nonhuman proteins^7,10^, despite known species- and cell-type-specific localization patterns^15^. Finally, models are generally assessed on a minimal subset of all human proteins^8,9^. For example, DeepLoc2 was evaluated on 1,717 proteins, covering just 8.5% of the human proteome.

To address these limitations, we developed a more rigorous and comprehensive benchmark for sequence-based subcellular localization prediction. We first defined a three-level hierarchical label set that captures both coarse- and fine-grained compartments, enabling biologically meaningful multilabel classification. Using this scheme, we resolved discrepancies in the content and granularity of localization annotations from UniProt, the Human Protein Atlas (HPA) and OpenCell. By integrating these resources, we constructed a unified training set and a test set that includes 3,814 human proteins with high-confidence localization data, more than twice the size of the DeepLoc2 benchmark. We used these datasets to retrain and re-evaluate several published models and systematically test combinations of PLMs and feature aggregation strategies. Our benchmarking plan can be found in stage 1 of our Registered Report at https://springernature.figshare.com/articles/journal_contribution/A_comprehensive_benchmark_of_sequence-based_subcellular_localization_predictors_for_human_proteins/31282576.

To assess biological utility, we evaluated the top-performing model in three exploratory tasks: motif identification via attention profiles, multimodal learning with protein–protein interaction (PPI) networks, and prediction of localization changes for pathogenic missense variants. In all cases, performance remained limited. Even the best model struggled with rare compartments and failed to generalize to mislocalizing variants. Our results reveal fundamental shortcomings in current approaches and highlight the key challenges in developing the next generation of accurate and robust localization predictors.

## Results

### Integrating protein databases and constructing train and test sets

**Fig. 1:**
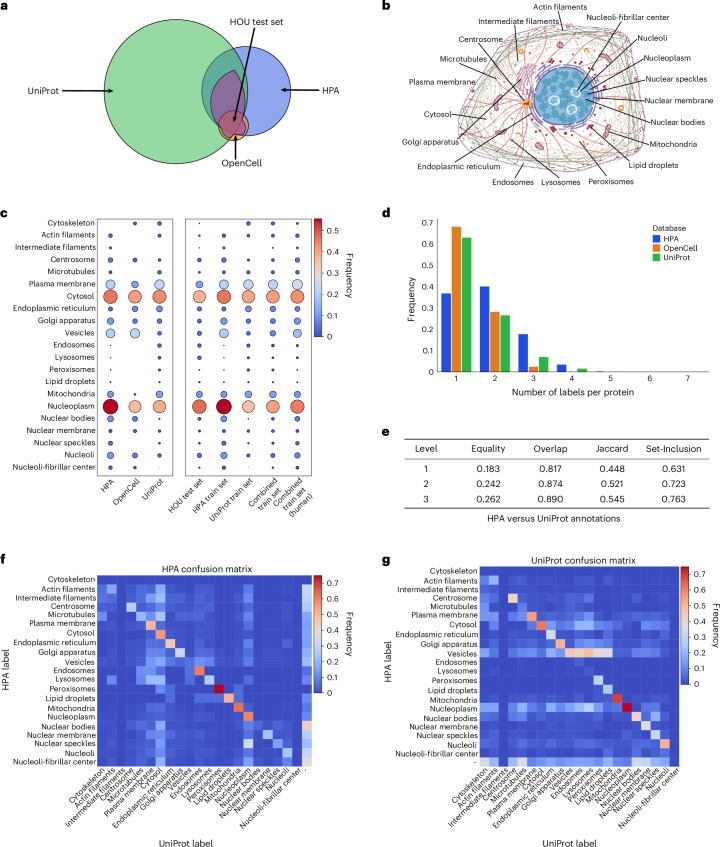
Construction of training and high-confidence test sets from multiple protein databases. **a**, Venn diagram showing how the HOU test set was derived from database overlap. **b**, Schematic of subcellular compartments in level 1 of the hierarchical label set. **c**, Level 1 label frequency for each database and constructed dataset. **d**, Histogram of multilocalization frequencies per protein across databases. **e**, Annotation agreement statistics between HPA and UniProt. **f**,**g**, Confusion matrices between HPA annotations and UniProt annotations normalized by HPA label frequency (**f**) and normalized by UniProt label frequency (**g**).

In this study, we sourced localization annotations from three widely used databases: UniProtKB^18^, the Human Protein Atlas (HPA)^15^ and OpenCell^19^ (Fig. 1a). UniProt provides detailed information on protein sequences, functions, interactions, and post-translational modifications. We focused on SwissProt, the manually curated subset containing over 500,000 proteins, and retained only eukaryotic entries with localization annotations explicitly supported by experimental evidence. The subcellular section of the HPA contains antibody-based immunofluorescence images for more than 13,000 human proteins across 37 cell lines, with manual annotations of localization patterns by domain experts. OpenCell similarly provides high-quality image-based annotations, profiling 1,310 endogenously tagged proteins in HEK293T cells using CRISPR-engineered fluorescent labeling. Like HPA, OpenCell’s annotations are derived from fluorescence microscopy, but are limited to a single cell type.

Integrating these resources presented two major challenges, both linked to variation in label granularity. First, label vocabularies vary greatly: UniProt, drawing heavily from diverse literature sources, uses over 300 distinct localization terms, while OpenCell, constrained by imaging resolution, uses only 17 compartments. Second, even when databases share similar labels, they often assign slightly different compartments to the same protein. Thus, annotations can differ in both identity and specificity. Such discrepancies can arise from both biological and technical factors: protein localization can be cell-type specific^15^, and different experimental methods introduce distinct biases. For example, imaging facilitates the detection of multilocalization compared to biochemical approaches. Antibodies can be unspecific, as has been reported in HPA, and genetically tagged proteins can mislocalize^20^. Additionally, not all compartments are amenable to fluorescent tagging, which was used in OpenCell^19^.

To aggregate localization annotations across sources, we defined a three-level hierarchical label set of cellular compartments arranged by decreasing specificity (Supplementary Table 1). Level 1 contains 21 fine-grained compartments, covering most aspects of cell architecture (Fig. 1b). We map annotations from each database to this standard label set (Methods), enabling systematic evaluation of localization predictors at varying resolutions.

Further, standardizing labels allowed us to quantify differences in frequency of and disagreement in localization annotations between databases. The frequency of individual compartment labels varies considerably between resources (Fig. 1c and Supplementary Tables 2–4). For example, UniProt lacks labels for intermediate filaments and the nucleoli-fibrillar center, and HPA and OpenCell have a larger frequency of vesicle-localizing proteins than UniProt. Another major source of disagreement relates to protein multilocalization. HPA reports multiple compartments for 62.9% of level 1-annotated proteins, compared to 36.7% in UniProt and 31.6% in OpenCell (Fig. 1d). This is reflected in the fact that among the proteins shared by HPA and UniProt, fewer than 27% have identical localization label sets, yet more than 80% share at least one compartment label (Fig. 1e).

To examine how label granularity affects agreement, we constructed confusion matrices between HPA and UniProt (we excluded OpenCell due to its lower protein coverage). We observed broad agreement for major compartments (for example, plasma membrane, cytosol, endoplasmic reticulum (ER), mitochondria and nucleoplasm) but clear differences for fine-grained compartments. HPA’s granular cytoskeletal categories (for example, intermediate filaments, microtubules) are often recorded as cytosol or omitted entirely in UniProt (Fig. 1f). Likewise, HPA’s nuclear substructure labels (for example, nuclear bodies and nucleoli-fibrillar center) are frequently absent in UniProt, and nuclear speckles are typically labeled more generally as nucleoplasm (Fig. 1f). Conversely, UniProt distinguishes lysosomes, endosomes, peroxisomes and lipid droplets, whereas HPA often groups these into a single ‘vesicles’ label (Fig. 1g). Additionally, we identify 583 proteins with no common level 3 localization annotations between HPA and UniProt. We provide a list of these proteins as a Supplementary Data File and encourage further investigation of their true localization. Despite these differences in granularity, the labels are largely consistent in a biological sense for a majority of human proteins.

To address label inconsistencies, we curated a high-confidence benchmark test set, termed the HPA-OpenCell-UniProt (HOU) test set, consisting of proteins with at least one localization label independently supported by two of the three databases (Fig. 1a). This conservative intersection yields a test set enriched for reliable localization labels, enabling robust and unbiased model evaluation. The remaining proteins from HPA and UniProt were used to construct a fivefold cross-validation training set, with sequence similarity less than 40% identity, both between the train and test sets and across training folds (Methods). These datasets support fair, detailed evaluation of multilabel localization predictors, establishing a new benchmarking standard for the field.

### Benchmarking existing published sequence-based localization predictors

**Fig. 2:**
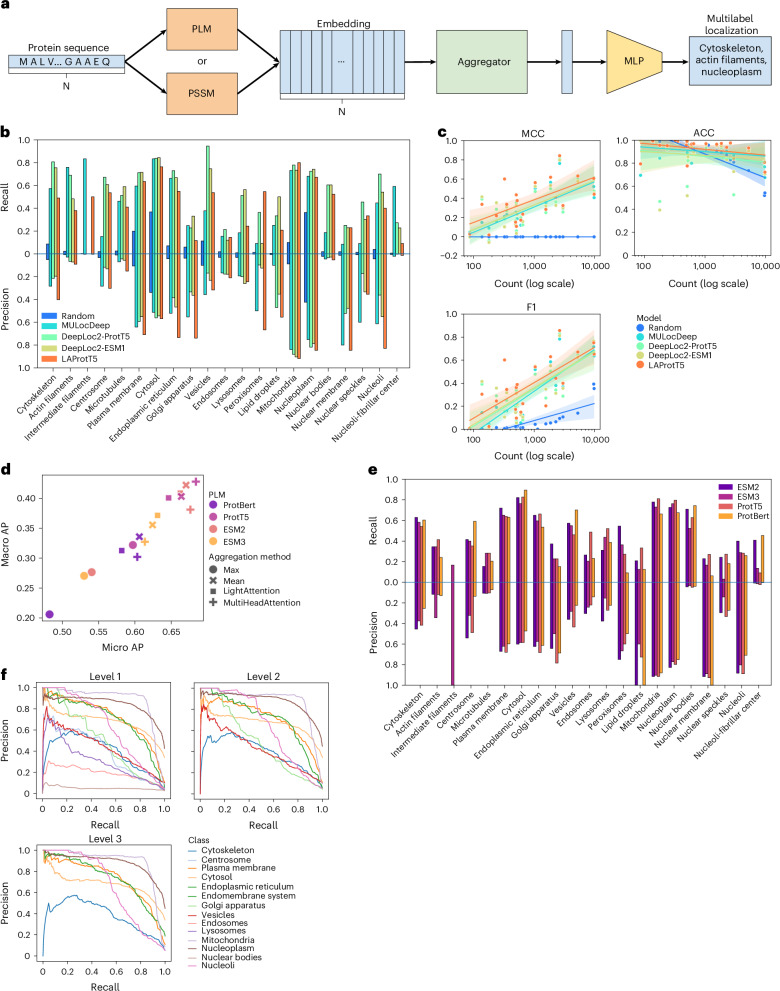
Benchmarking the performance of existing baseline models and systematic evaluation of PLM and aggregation method combinations. **a**, General architecture for protein sequence to localization models. **b**, Bar plot of per-class precision and recall of baseline models on the HOU test set. Recall shown on positive *y* axis and precision shown on negative *y* axis. **c**, Scatter plot relating class size (in training set) and model performance, measured by MCC, accuracy and F1 score, for each baseline model. Line of best fit shown for each model with the 95% confidence interval shaded (*n* = 21 for each line). **d**, Scatter plot of macro and micro AP for all PLM and aggregation method combinations. **e**, Bar plot of per-class precision and recall of best PLM models from systematic evaluation on the HOU test set. Recall shown on positive *y* axis and precision shown on negative *y* axis. **f**, PR curves for ProtT5-MHA model across common compartments and all label levels.

Models predicting subcellular localization from protein sequences typically share a common architecture (Fig. 2a), encoding sequence information via PLMs or position-specific scoring matrices (PSSMs)^21^, aggregating residue-level features, then classifying aggregated embeddings with a multilayer perceptron (MLP). We benchmarked three state-of-the-art models, DeepLoc2 and LAProtT5 (PLM-based) and MULocDeep (PSSM-based), which were selected for their documentation quality and ease of use (Supplementary Table 5). These models were trained and evaluated on our curated datasets, alongside a Bernoulli random baseline (Methods). Performance was assessed at all levels of our hierarchical label set, and we report both averaged and per-compartment metrics (Supplementary Tables 6–9).

Performance improved as label granularity decreased (Table 1). All models excelled on major membrane-bound organelles (for example, plasma membrane, ER and mitochondria) (Fig. 2b); however, performance declined for cytoskeletal compartments and certain nuclear substructures, including nuclear bodies and the nucleoli-fibrillar center. The model also performs well on the two most common compartments, cytosol and nucleoplasm, but even the random baseline achieved moderate scores on these two compartments, highlighting how label frequency can inflate performance metrics.

**Table 1:** Average performance of all models

Model	Accuracy			Macro AP			Micro AP			Num labels		
	1	2	3	1	2	3	1	2	3	1	2	3
Random	0.050	0.068	0.071	–	–	–	–	–	–	**1.613**	1.468	1.443
MuLocDeep	0.227	0.354	0.391	0.290	0.552	0.634	*0.590*	0.672	0.687	2.341	1.489	1.474
DeepLoc2-ProtT5	0.078	0.334	0.448	0.332	0.584	0.669	0.401	0.672	0.718	3.514	1.755	1.397
DeepLoc2-ESM1	0.109	0.356	0.453	0.324	0.590	0.662	0.423	0.671	0.703	3.300	1.612	1.404
LAProtT5	*0.238*	*0.407*	*0.512*	*0.371*	*0.614*	*0.682*	0.582	*0.735*	*0.755*	1.910	*1.392*	*1.225*
ESM2	0.232	0.458	0.518	0.422	**0.651**	**0.717**	0.670	0.746	0.780	2.109	**1.255**	1.218
ESM3	0.245	0.402	0.476	0.372	0.587	0.667	0.631	0.708	0.740	2.007	1.329	**1.261**
ProtT5	**0.272**	**0.476**	**0.537**	**0.428**	0.649	0.709	**0.684**	**0.748**	**0.787**	*1.852*	1.314	1.193
ProtBert	0.122	0.281	0.431	0.336	0.565	0.649	0.606	0.681	0.716	3.058	1.727	1.327

Table of averaged metrics for existing baseline models (top) and best-performing systematic-PLM models (bottom) trained at each level of the hierarchical label set. For each level and metric, the best result for baseline and systematic-PLM are emphasized. The best result is bolded and the second-best result is italicized.

**Extended Data Fig. 1:**
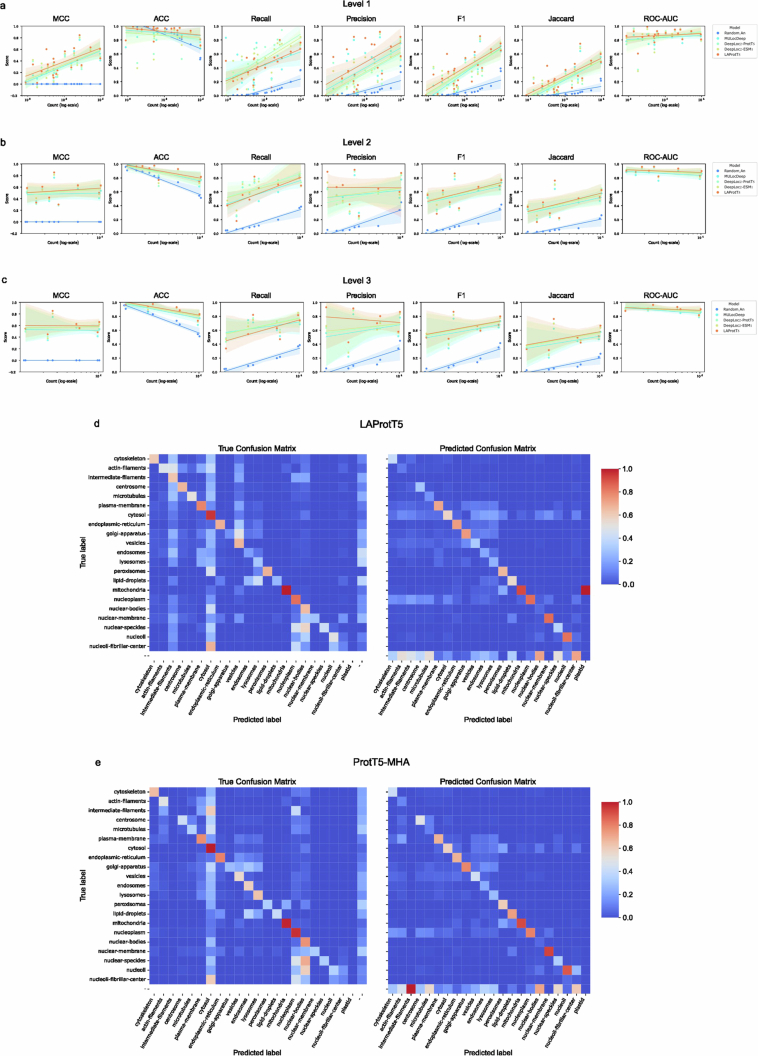
Performance metrics and confusion matrices of best models. Scatter-plot relating class frequency (in training set) and model performance for each baseline model. Line of best fit shown for each model with the 95% confidence interval **a**, level 1 (n=21), **b**, level 2 (n=9) and **c**, level 3 (n=7) of the hierarchical label set. Heatmap of confusion matrix for **d**, LAProtT5 model and **e**, ProtT5-MHA model trained on level 1 labels (left: normalized by the frequency of true labels, right: normalized by frequency of predicted labels). See Methods section for details about construction of confusion matrix for mulitlabel prediction.

To fully assess the impact of label frequency on model performance, we plotted per-compartment evaluation metrics against compartment class size (Fig. 2c). Metrics such as F1 score improved with class size, whereas accuracy decreased, as high accuracy on rare compartments can result from consistently predicting negatives. The random baseline’s performance similarly correlated with class size across most metrics (Extended Data Fig. 1a–c), except for Matthews correlation coefficient (MCC), which remained stable. Linear regression models quantified this dependence via *R*^2^ values (Supplementary Table 10). F1 score showed high sensitivity to label frequency, whereas MCC demonstrated robustness, underscoring its value as a balanced metric for multilabel localization evaluation despite being less intuitive to interpret.

Among the benchmarked models, LAProtT5 consistently achieved the highest performance across most metrics (Supplementary Table 6), demonstrating notably high precision on large compartments such as the ER, nuclear membrane and nucleoli (Fig. 2c). This precision corresponds to a lower average number of predicted compartments per protein; LAProtT5 averaged fewer than two level 1 labels per protein, reflecting a more conservative prediction strategy, in contrast to DeepLoc2-ProtT5, which assigned an average of 3.5 labels per protein. To elucidate LAProtT5’s limitations, we analyzed its confusion matrix (Extended Data Fig. 1d; Methods contains matrix construction details). While accurately predicting major compartments, it struggled with rare classes. We noticed frequent misclassifications between nuclear speckles and nuclear bodies, lipid droplets and lysosomes, and the Golgi apparatus and vesicles (Extended Data Fig. 1d). Additionally, the model often failed to identify lysosomes, endosomes, and lipid droplets when present. Furthermore, we observe occasional false positives in nuclear bodies, nucleoli, nuclear speckles, nucleoli-fibrillar center, and cytoskeletal subcompartments (Extended Data Fig. 1d). These findings emphasize that current sequence-based models struggle to handle rare and fine-grained compartments.

### Systematically evaluating PLMs and aggregation strategies

In addition to benchmarking previously published methods, we evaluated several widely used PLMs on the task of subcellular localization prediction. We selected four pretrained models: ESM2 (ref. ^22^), ESM3-small-open^23^, ProtT5 (ref. ^24^) and ProtBert^25^. Each model generates per-residue embeddings, which we compressed into fixed-length representations using one of four aggregation methods: max pooling, mean pooling, light attention or multihead attention (MHA). All 16 possible model combinations were trained and evaluated using our new datasets with both focal^26^ and cross-entropy losses (Methods provides training details).

Compared to attention-based strategies, max pooling consistently underperformed, whereas mean pooling proved surprisingly effective, particularly for ESM2 (Fig. 2d). For each PLM, we report metrics using the aggregation method that achieved the highest macro-averaged precision (Supplementary Table 11). The top-performing combinations were ESM2 with mean pooling and ProtT5 with MHA, with the latter achieving the best performance across most averaged metrics and surpassing all previously published methods (Table 1).

Again, we report averaged and per-compartment metrics (Supplementary Tables 6 and 12–14) and find that performance trends mirrored those observed in the previously published models. All models achieved high precision and recall for common and well-annotated compartments, such as the plasma membrane, cytosol, mitochondria and nucleoplasm (Fig. 2e). In contrast, performance was lower for smaller or less frequently annotated compartments, including cytoskeletal substructures, nuclear bodies and the nucleoli-fibrillar center. Notably, all models except ESM3 failed to predict intermediate filaments for any protein, likely reflecting the scarcity of this label in the training set.

We selected the ProtT5 model, referred to as ProtT5-MHA, as the reference model for downstream analyses as it achieved the highest macro-averaged precision overall. To better characterize its performance, we visualized precision–recall (PR) curves for all classes with at least 50 samples in the HOU test set across all hierarchical levels (Fig. 2f). PR curves for mitochondria and nucleoplasm approached the ideal top-right corner, indicating high precision and recall across thresholds. In contrast, PR curves for endosomes and lysosomes revealed poorer performance, with low precision across all recall thresholds. Notably, for labels that are identical across hierarchy levels, PR curves remained virtually unchanged. This suggests that the model’s predictions for these classes are robust and unaffected by changes in label granularity elsewhere in the hierarchy.

Finally, we examined the confusion matrix of the level 1 ProtT5-MHA (Extended Data Fig. 1e) model to identify common misclassifications. Its overall pattern closely resembled that of LAProtT5, with a few notable differences. ProtT5-MHA showed improved accuracy in predicting lipid droplets but lower accuracy for endosomes, lysosomes and nucleoplasm. It also failed to make any predictions for intermediate filaments, further highlighting the difficulty of learning rare classes even for high-performing models. Our systematic benchmarking shows that careful pairing of language models and aggregation strategies can substantially improve localization prediction, offering a principled path for future model design.

### Analyzing performance stratified by protein properties

**Extended Data Fig. 2:**
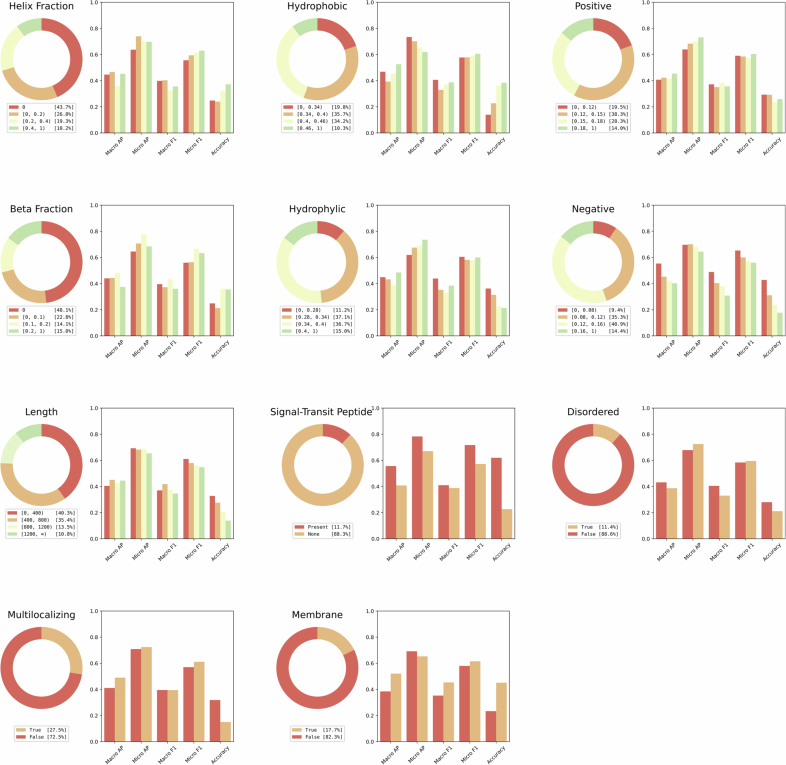
Performance stratified by all protein properties. Performance of ProtT5-MHA on HOU proteins stratified by protein properties. Circle plots of frequency of stratified bins and barplots of stratified performance measured by Macro AP, Micro AP, Macro F1, Micro F1 and accuracy.

To assess whether model performance varies with structural, sequence, or physicochemical properties of the proteins, we evaluated ProtT5-MHA on stratified subsets of the HOU test set. Proteins were grouped into discrete bins, four quartiles for continuous properties or two categories for binary annotations (Methods), and performance in each bin was measured separately. Across most features, variation in performance was modest, and bin rankings often differed between metrics (Extended Data Fig. 2), reflecting both metric-specific biases (Supplementary Note) and random effects from small sample sizes. Nonetheless, several notable trends emerged.

**Fig. 3:**
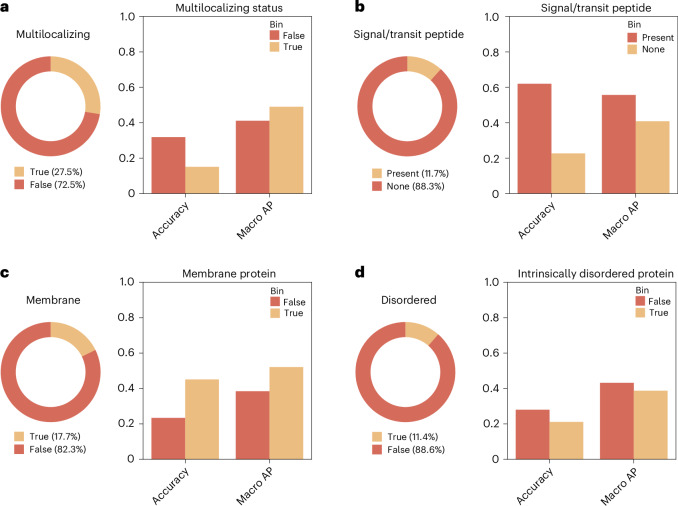
Localization prediction performance stratified by protein properties. Model accuracy and macro AP stratified by structural or sequence properties in the HOU test set. **a**, Properties include number of level 1 labels (single versus multilocalizing proteins). **b**, Presence of signal or transit peptides. **c**, Intramembrane or transmembrane proteins. **d**, Intrinsically disordered proteins.

Proteins annotated with a single subcellular location were predicted with higher accuracy than those with multiple localization labels (Fig. 3a); however, somewhat counterintuitively, the macro-averaged average precision (AP) was higher for multilocalizing proteins. This likely reflects the fact that multilocalizing proteins often have a primary compartment in a large, well-represented class (for example, nucleus or cytosol) that is predicted reliably, which boosts AP even if the secondary compartment is missed.

Unsurprisingly, the presence of signal or transit peptides substantially improved model performance (Fig. 3b), consistent with their role as strong, evolutionarily conserved localization determinants to the mitochondria, nucleus and secretory pathways.

Structural context also influenced predictions. Membrane-associated proteins achieved higher accuracy and macro AP (Fig. 3c), suggesting that recurring structural motifs such as transmembrane helices may facilitate classification. In contrast, intrinsically disordered proteins were harder to classify by both accuracy and macro AP (Fig. 3d). This is consistent with their functional diversity^27^ and potentially dynamic localization patterns.

Overall, stratified analyses indicate that conserved sequence motifs and structural features enhance localization prediction. Future development could improve prediction performance by directly incorporating structural data or adopting model architectures that are better suited to capture these structural features.

### Evaluating biologically relevance of attention profiles

**Fig. 4:**
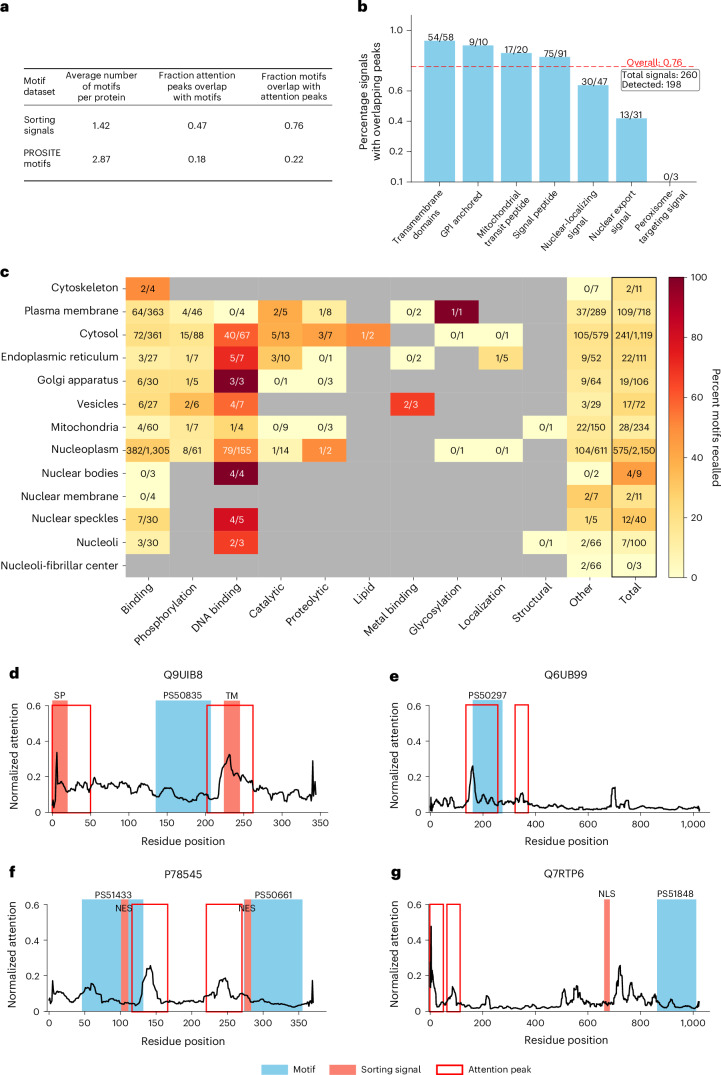
Overlap of attention peaks and known sequence motifs. **a**, Statistics summarizing frequency at which attention peaks and known motifs and sorting signals overlap. **b**, Bar plot of rate at which attention peaks overlap different types of sorting signals. **c**, Heatmap of rate at which attention peaks overlap with PROSITE motifs, stratified by protein localization label. Text shows the fraction of PROSITE motifs recalled. **d**–**g**, Examples of normalized attention profiles with labeled peaks, motifs and sorting signals for four proteins (UniProt IDs Q9UIB8 (**d**), Q6UB99 (**e**), P78545 (**f**) and Q7RTP6 (**g**)).

Next, we asked whether ProtT5-MHA attends to biologically meaningful sequence motifs. We examined two sources: sorting signals curated by DeepLoc2 (ref. ^9^), available for 184 proteins in our HOU test set, and motifs from the PROSITE database^28^ (Methods). ProtT5-MHA attends strongly to sorting signals, with 50% of attention peaks overlapping annotated signals and 76% of all signals detected by at least one peak (Fig. 4a). In contrast, overlap with PROSITE motifs was much lower, with 18% of all attention peaks overlapping with PROSITE motifs and only 22% of motifs detected, which is consistent with the fact that most PROSITE motifs are not directly related to localization.

ProtT5-MHA reliably detected nearly all types of sorting signals (Fig. 4b), with the exception of peroxisome targeting signals. This may reflect the rarity of peroxisomes in the training set or the tendency of their signals to occur at sequence termini, which are not always captured by our peak-calling method (Methods).

Compared to sorting signals, PROSITE motifs provided weaker signals, yet the model consistently attended to DNA-binding domains, likely reflecting their correlation with nuclear localization (Fig. 4c). ProtT5-MHA also focused on some metal-binding motifs in vesicle-localizing proteins. We hypothesize that these correspond to lysosomal proteins, although lysosome-specific labels are absent from the HOU test set. Together, these findings highlight that even functional or binding motifs not explicitly tied to localization can still provide informative signals to localization predictors.

Finally, qualitative inspection revealed examples where attention peaks aligned almost perfectly with known sorting signals (Fig. 4d) or PROSITE motifs (Fig. 4e). In other proteins, attention peaks focused on flanking regions (Fig. 4f) or entirely different sequence segments (Fig. 4g). Such unexplained peaks may reflect sequence motifs or structural features not captured by our motif annotations.

**Extended Data Fig. 3:**
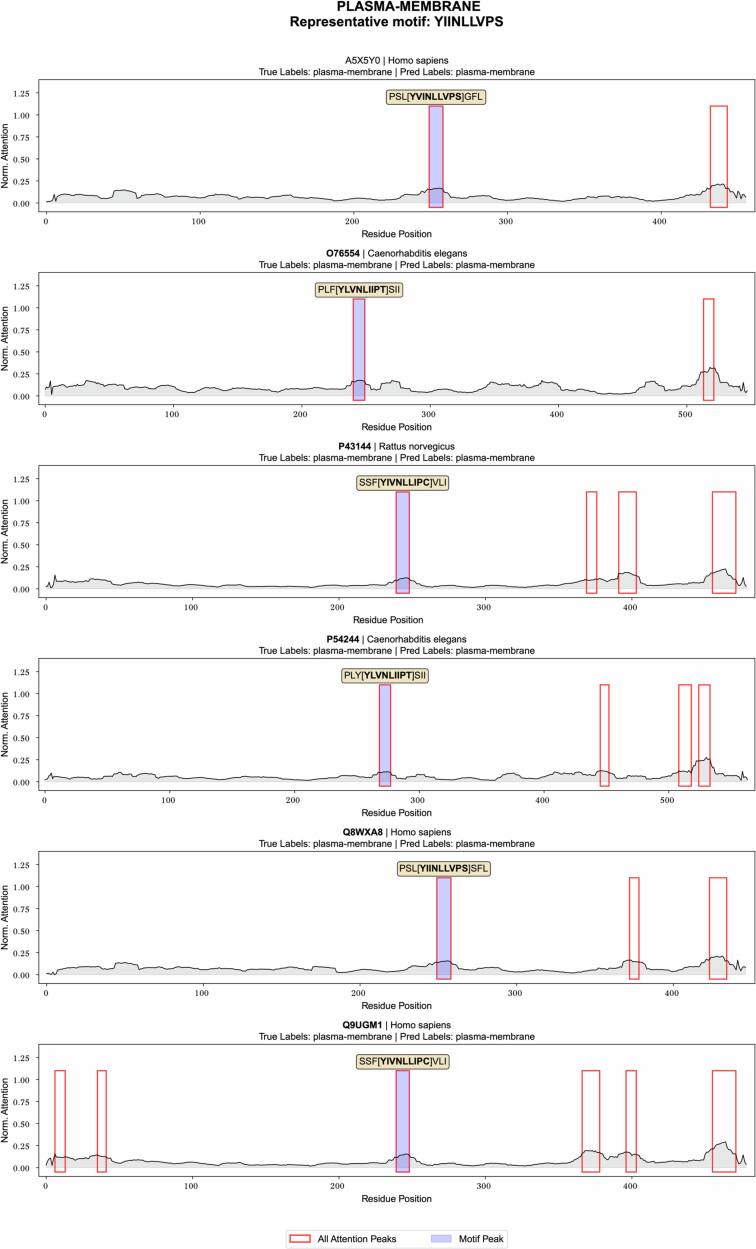
Attention peak across species for plasma-membrane motif. Normalized attention profiles for homologous plasma-membrane localizing proteins across species with conserved ‘fuzzy’ motif identified by model attention peak.

**Extended Data Fig. 4:**
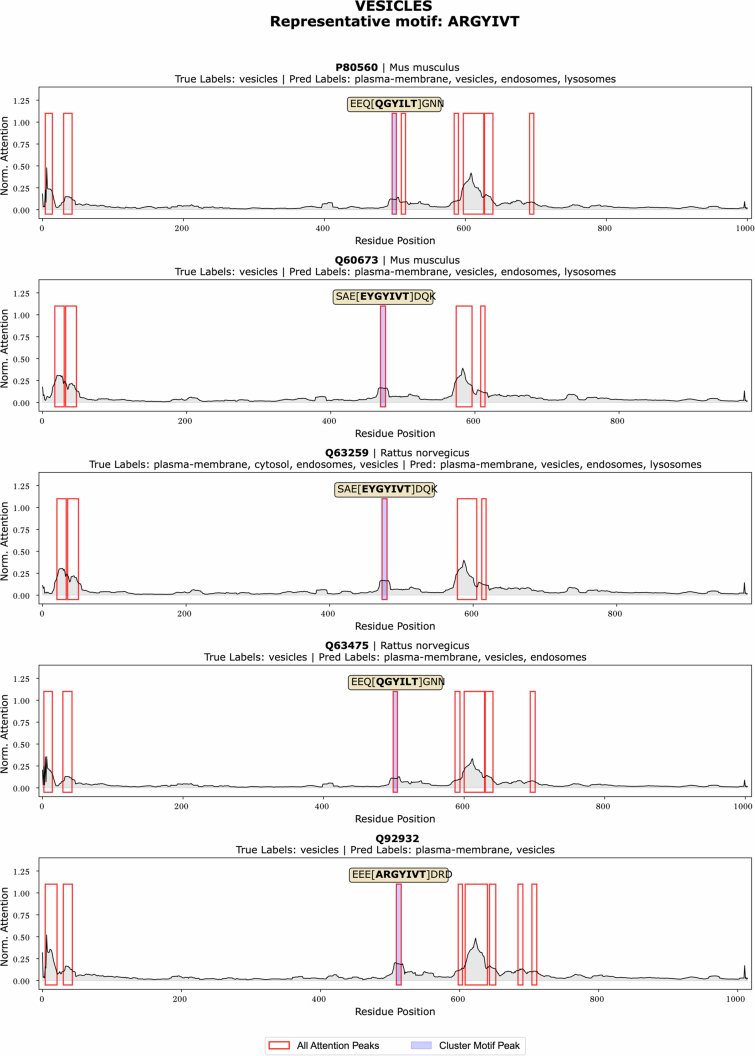
Attention peak across species for plasma-membrane motif. Normalized attention profiles for homologous vesicle localizing proteins across species with conserved ‘fuzzy’ motif identified by model attention peak.

To investigate further whether attention peaks picked up on any new sorting signals, we isolated subsequences captured by attention peaks for unannotated regions of proteins which ProtT5-predicts at least one correct localization. Clustering motifs per localization compartment revealed nine fuzzy motifs of interest that appear across three or more species (Supplementary Table 15). For example, the fuzzy motif, ‘YIINLLVPS’ can be seen in plasma-membrane-localizing homologs across a number of species (Extended Data Fig. 3). We hypothesize that this sequence motif is identified by the model because of its high frequency of hydrophobic residues. However, we identified other fuzzy motifs across species, such as ‘ARGYIVT’ in vesicle-localizing proteins, for which it is less clear why the model highlights this region (Extended Data Fig. 4). Future work could further investigate unannotated attentions motifs by determining whether they correlate to preserved structural motifs or investigating the effects of mutations.

### Incorporating PPI data into localization predictors

Proteins must be in close proximity to interact. Thus, PPI networks may carry implicit signals about subcellular localization that could inform localization predictors. Some past works have attempted to predict localization from PPI networks^29–31^ among other sources of information, but none have tried to combine both PPI networks and PLM embeddings. To investigate this avenue, we augment our best-performing model with GraphSage^32^, a type of graph convolution network, to combine sequence and PPI data (Methods). We evaluate the resulting model, ProtT5-MHA-PPI, in two inference modes: with and without PPI network edges. We expected that message-passing through PPI edges would amplify localization signals when interactors shared the same compartment.

**Fig. 5:**
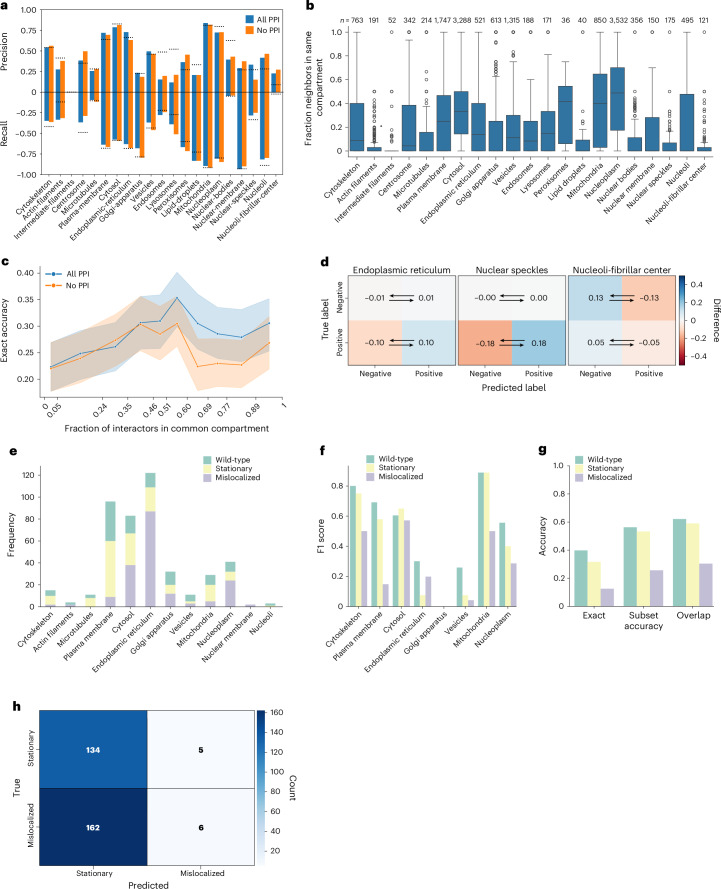
PPI integration and model performance on predicting mislocalization of pathogenic protein variants. **a**, Per-class precision and recall of ProtT5-MHA-PPI, with ProtT5-MHA performance shown as a black dotted line. Results are shown for inference with PPI edges (blue) and without edges (orange). **b**, Box-and-whisker plots showing, for each compartment, the fraction of STRING interactors that share the same compartment as the protein. Proteins are taken from both the combined training and test sets and restricted to those with at least one interactor in STRING. Box plots show the median (center line), 25th and 75th percentiles (box bounds), and whiskers extending to 1.5× the interquartile range. Points beyond the whiskers are shown as outliers. Sample sizes (*n*) are displayed above each box. **c**, Line plot of exact accuracy versus fraction of interactors. Proteins were grouped into ten bins based on the fraction of their interactors sharing at least one compartment label, with bins balanced to contain a similar number of proteins. For each set of binned proteins, we display the average per-sample accuracy with the 90% confidence interval shaded. **d**, Difference in confusion matrices for ProtT5-MHA-PPI with and without PPI edges, shown for three compartments. Arrows indicate shifts in predictions, either from false negatives to true positives or from false positives to true negatives. **e**, Frequency of level 1 subcellular compartments for wild-type proteins and stationary or mislocalized variant proteins. **f**, F1 scores for ProtT5-MHA predictions of wild-type, stationary and mislocalized proteins. **g**, Accuracy of ProtT5-MHA predictions for wild-type, stationary and mislocalized proteins. Accuracy calculated as exact match, subset or nonzero intersection of true and predicted labels. **h**, Confusion matrix for ProtT5-MHA predicting variant proteins as stationary or mislocalized.

**Extended Data Fig. 5:**
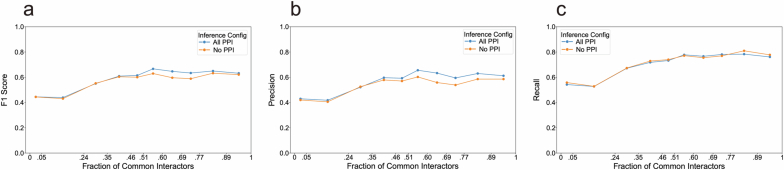
Impact of PPIs on prediction performance. Proteins were grouped into ten bins based on the fraction of their interactors sharing at least one compartment label, with bins balanced to contain a similar number of proteins. Line plot of average **a**, F1 score, **b**, precision, and **c**, recall for proteins in each bin versus fraction of interactors.

However, ProtT5-MHA-PPI did not substansially outperform ProtT5-MHA by any average metric (Supplementary Table 16), nor did it dramatically or consistently improve performance on individual compartments (aside from peroxisomes, a very small class) (Fig. 5a). When combining PPI data with our localization annotations, we find that the median fraction of interactions occurring in the same compartment is less than 0.5 for nearly all classes (Fig. 5b). This could be because PPI networks tend to be noisy and incomplete, or because interactions frequently happen at the interface of cellular compartments (for example, between the nuclear membrane and cytosol). In line with this, we find that including PPI edges during inference only boosts accuracy for proteins with a high proportion of interactors localizing to a common compartment (Fig. 5c). We observe a similar trend with F1 scores, driven by an improvement in precision rather than recall (Extended Data Fig. 5a–c). This suggests that PPI information can help control false positive predictions when message-passing transmits a consistent localization signal from interactors.

**Extended Data Fig. 6:**
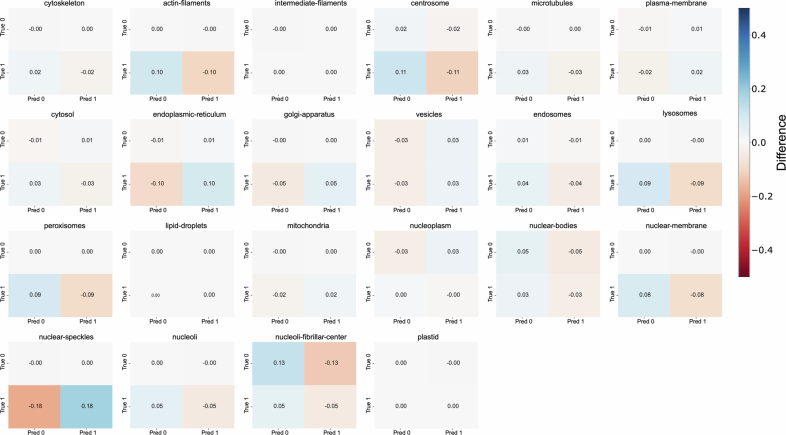
Impact of PPI on prediction performance per compartment. Difference in confusion matrices for ProtT5-MHA-PPI with and without PPI edges, shown for all compartments. Predictions shift between true positive and false negatives and between true negatives and false positives.

By visualizing the difference in confusion matrices when using versus not using PPI edges during inference, we identified just three compartments for which PPI data clearly improve predictions (Fig. 5d). For the ER, the number of true-positive predictions increased by 10% (16 additional proteins predicted positive). Similarly, for nuclear speckles, the number of true-positive predictions increased by 18% (ten additional samples predicted positive). Inversely, for the nucleoli-fibrillar center, including PPI edges reduced the false-positive rate, increasing the number of true negative predictions by 13% (551 more correct true negatives, albeit 66 additional false positives; Extended Data Fig. 6 and Supplementary Table 17).

In summary, PPI networks seem too noisy to consistently boost the performance of sequence-based predictors. They may enhance performance for select compartments or when many interactors share the same localization pattern. Our exploratory investigation, which used a straightforward graph extension of ProtT5-MHA, was not designed to fully exploit such cases. Future work could employ more sophisticated multimodal architectures better suited to leverage sparse, noisy PPI networks.

### Assessing model generalization to pathogenic protein variants (exploratory analysis)

To assess whether state-of-the-art sequence-based localization models can detect mislocalization effects of pathogenic mutations, we evaluated ProtT5-MHA on experimentally profiled protein variants from Lacoste et al.^33^. This study systematically characterized the subcellular localization of over 3,000 missense variants across more than 1,000 genes, finding that approximately one-sixth of pathogenic variants are mislocalized. The authors concluded that most mislocalization events result from protein instability or disrupted membrane insertion rather than altered sorting signals or trafficking-related protein–protein interactions.

After applying a series of filters on the variants studied (Methods), we retained 103 genes and 308 variants from the Lacoste dataset. Of these, 169 were classified as mislocalized and 139 as stationary (localized identically to the corresponding wild-type proteins). For comparison, we also evaluated the unmutated (wild-type) protein sequences for the same genes. A major bottleneck in our filtering process stemmed from mismatches between the description of mutations and our amino-acid sequences, indicating that the specific isoforms used by Lacoste et al. differ from the canonical isoform chosen by UniProt. This underscores an often-ignored challenge in designing sequence-based predictors: localization can be isoform and cell-type-specific.

Initial comparison of compartment frequencies across wild-type, stationary, and mislocalized proteins revealed an enrichment of mislocalized variants in the ER (Fig. 5e). This observation aligns with the findings of Lacoste et al., finding that many disease-associated mutations cause misfolding and retention in the ER by quality control mechanisms. Next, we assessed ProtT5-MHA’s predictive accuracy across these groups. The model achieved high F1 scores for wild-type proteins, and moderately lower scores for stationary variants, but performance dropped substantially for mislocalized variants across nearly all compartments, and particularly for the plasma membrane (Fig. 5f). Overall label accuracy comparisons confirmed this pattern (Fig. 5g).

To investigate the poor performance of the model on mislocalized variants, we examined how often it changed its localization prediction in response to mutations. Notably, the model altered its prediction for only 11 out of 308 variant sequences, indicating strong insensitivity to single-residue changes (Fig. 5h). This may reflect overfitting, or more likely, support the idea that mislocalization is often driven by protein instability or membrane insertion, factors not readily captured by current sequence-based models.

In summary, ProtT5-MHA does not generalize well to disease-related protein variants. Its limited ability to detect mutation-induced mislocalization likely reflects the fact that localization is influenced by factors beyond primary sequence, such as protein folding, stability and cellular context. These findings suggest that variant-aware training and the integration of structural or stability predictors may be necessary to improve clinical applicability.

## Discussion

In this study, we conducted a comprehensive benchmark of sequence-based subcellular localization predictors, systematically evaluating published methods, PLMs and aggregation strategies. To assess the models rigorously, we integrated annotations from three major protein databases and constructed a highly validated test set of multilabel localization annotations for human proteins (HOU localization test set). Beyond benchmarking, we explored how model attention correlates with known sequence motifs, investigated the integration of PPI networks and assessed model performance on pathogenic missense variants known to mislocalize. Although sequence-based localization prediction has largely been considered a solved problem, our findings tell a different story. Even the best model shows limited performance on multilocalizing proteins and rare subcellular compartments, and fails to generalize to mislocalized variants, revealing substantial room for improvement.

Based on our analyses, we identify several concrete recommendations for building more accurate and generalizable localization predictors. While our best model seems to have implicitly learned to attend to some sorting signals, future models may benefit from explicitly guiding attention to an expanded set of localization signals and function-related sequence motifs, as carried out by DeepLoc2 (ref. ^9^). Alternatively, using information beyond amino-acid sequences may necessary to meaningfully improve subcellular localization predictors. Future studies attempting to incorporate PPI data could adopt flexible architectures that selectively integrate interaction information. Then, interactions could boost the signal for certain compartments or for certain proteins whose interactors colocalize. Finally, models that explicitly predict membrane insertion or structural stability may improve predictions for some membrane-bound compartments and pathogenic variants prone to mislocalization. Collectively, these strategies highlight one of our main takeaways: unannotated sequences alone are often insufficient to determine localization at a fine-grained resolution, and predictors may benefit from additional input information, such as motifs, interactions and structural features.

All these suggestions aim to add auxiliary information to protein representations after encoding sequences with a PLM; however, a broader and more ambitious path forward would be to develop a next-generation protein representation model that co-embeds multiple types of data simultaneously. Rather than treating stability, localization and interactions as isolated post-training benchmark tasks, a multimodal protein representation model could learn directly from these diverse sources of biological signal. Achieving this will require extensive data curation and careful architectural choices designed to handle noisy and incomplete input, perhaps by adaptively prioritizing different sources of information.

Yet, no matter how much localization predictors improve, current approaches, and even our own benchmark, rest on several simplifying assumptions that overlook key biological realities. First, studies typically assume that protein sequences are unique, even though most proteins have multiple isoforms. Further, the choice of a ‘canonical sequence’ is often arbitrary and differs between databases. This becomes an issue when isoforms have distinct localization patterns, which has been observed in some cell-types^34–36^. Second, all current approaches assume that localization is fixed, even though it is well known that localization patterns can vary over cell states (for example, the cell cycle) and cell types^15,37^. Last, framing localization prediction as a multilabel classification task compresses the spatial gradient of localization patterns captured by microscopy. Sequence-to-image models, like CELL-Diff^38^ and PUPS^39^, offer a more faithful representation, modeling protein localization as a distribution over cellular compartments rather than a set of discrete labels.

An additional avenue for advancing localization prediction is to consider local translation. Although RNA localization has not been systematically characterized across all genes, growing evidence suggests that local translation is more widespread than previously appreciated^40–42^. One mechanism attracting increasing attention is the role of biomolecular condensates, which concentrate mRNAs and RNA-binding proteins to regulate localized translation and other processes^43–45^. Indeed, up to 20% of human proteins are thought to dynamically associate with condensates^46^. Given their involvement in signaling^47^, transcriptional activation^48^ and disease^49^, predicting condensate association, as carried out in ProtGPS^50^, could be highly impactful. Looking forward, we see two promising directions: (1) applying emerging DNA and RNA language models, such as Evo2 (ref. ^51^) and RiNALMo^52^, to predict mRNA localization from 3’ untranslated region ‘zip-codes,’ and (2) extending localization predictors to include condensate association of both mRNAs and proteins. Together, these strategies could expand predictors beyond fixed compartments to include dynamic and transient cellular environments.

Protein localization is fundamentally complex and dynamic. Capturing it accurately will require reframing computational approaches to account for isoform diversity, cell-type specificity, spatial continuity and temporal dynamics. Furthermore, a truly mechanistic understanding of protein localization may require models with highly attuned attention mechanisms that are capable of identifying novel sorting signals and predicting the effects of mutations. By highlighting the limitations of current methods and the restrictive assumptions they rely on, we aim to revitalize interest in subcellular localization prediction as an open and pressing challenge. We call for renewed efforts in data collection, benchmark standardization and model development.

## Methods

### Localization datasets

In this study, we used three databases to construct our datasets: HPA v24, SwissProt from UniProt (release 2024_06), and OpenCell. We applied a series of filtering steps to the raw data from each source. The HPA assigns every protein a ‘Gene Reliability’ label reflecting the fidelity of antibody staining, and we include only those labeled as ‘Enhanced,’ ‘Supported’ or ‘Approved.’ For UniProt, we selected proteins based on the following criteria: they are (1) classified as ‘Reviewed’; (2) derived from eukaryotic organisms (taxonomy ID 2759); (3) not fragments; (4) not encoded in mitochondria, chloroplasts or plastids; and (5) annotated with at least one experimentally verified subcellular localization (evidence code ‘ECO:0000269’). OpenCell subcellular localization annotations are categorized into three grades, with grade 3 indicating the most prominent localization, grade 2 representing a weaker signal and grade 1 being ambiguous. We used grade 3 annotations in the construction of the training and test sets. For all proteins in all datasets we use the canonical isoforms as defined by UniProt. Based on these isoforms, we filtered out proteins that do not start with methionine or have fewer than 40 residues.

### Variant dataset

We obtained localization data for wild-type and variant proteins from Lacoste et al.^33^. The original dataset included 1,269 wild-type proteins and 3,445 variants, of which 250 were classified as mislocalized. For our analysis, we applied the following filtering steps: (1) the protein had at least one mislocalized variant; (2) a canonical UniProt sequence was available for the wild-type protein; (3) variant descriptions matched the UniProt canonical sequence (we excluded cases where the reported mutation referenced a residue that did not match the canonical sequence or implied a longer sequence than UniProt); (4) localization annotations mapped cleanly to level 1 of our hierarchical label set (proteins annotated with ‘foci’ or ‘rods and rings’ were excluded); and (5) we retained only wild-type proteins that had at least one corresponding variant remaining after the above filters. After filtering, 103 wild-type proteins and 308 variants remained, of which 169 were mislocalized. Of the 103 wild-type proteins, 45 were also present in the HPA-UniProt training set used to train ProtT5-MHA. These were retained due to the already limited size of the filtered dataset.

### PPI datasets

We used BioPlex^53^ and STRING^54^ databases to train the models with the PPI information. The BioPlex 3.0 database consists of around 118,000 interactions among 14,000 proteins in the HEK293T cell line. The STRING database consists of more than 6.86 M interactions of 20,000 proteins aggregated from various sources, and each interaction is given a ‘combined_score’, which is calculated by combining the probabilities from the different evidence channels. We used only the interactions that have a ‘combined_score’ >800 to include highly probable interactions in the analysis. This left us with 142,000 interactions among 13,000 proteins.

### Mapping localization categories

We mapped localization annotations from each database to level one of our standard set of subcellular compartments. Detailed mappings are provided in the codebase. We constructed HOU by first identifying the proteins that appear in at least two of the three databases and then taking the subset of proteins that have at least one localization label supported by at least two databases. HPA annotations are inconsistent in granularity for vesicle-like compartments, and UniProt annotations are inconsistent in granularity from cytoskeletal subcompartments. To address this discrepancy, we treat the ‘cytoskeleton’ and ‘vesicle’ labels as hierarchical within level 1 of our label set. Specifically, when a protein is annotated with ‘cytoskeleton’ by UniProt and as ‘microtubules’, ‘actin-filaments’, or ‘intermediate filaments’ by HPA, we take the more specific HPA annotation as the level 1 assignment. When a protein is annotated with ‘vesicles’ by HPA and ‘lysosomes’, ‘endosomes’, ‘peroxisomes’ or ‘lipid droplets’ by UniProt, then we take the more-specific UniProt label as the level 1 assignment.

For the sake of training and evaluating models, we add the higher level compartments labels ‘vesicles’ and ‘cytoskeleton’ whenever the annotation includes a sub-compartment. For example, if a protein in HOU is annotated with ‘lysosomes’ then in evaluation we consider the true label to be {‘lysosomes’, ‘vesicles’}; however, for the sake of defining multilocalization and evaluating the annotation agreement between HPA and UniProt we do not include the ‘vesicles’ and ‘cytoskeleton’ in addition to the sub-compartment annotations.

In the initial label mapping, we included the ‘plastid’ compartment because it represents a common localization annotation among eukaryotic proteins in UniProt. This inclusion allowed us to retain proteins annotated as localizing to plastids in combination with other compartments, which constituted approximately 20% of the plastid-associated proteins in the collected dataset. Incorporating the plastid label at this stage enabled the model to distinguish sequence signals associated with plastid targeting from those related to other subcellular compartments.

However, because the evaluation set consists exclusively of human proteins and because chloroplast proteins are in fact equally common and multilocalizing in UniProt as plastid proteins, we removed the plastid label from our proposed label set (Supplementary Table 1). Additionally, while the final datasets in our Git Repo contain the data that we trained our models on, which include the plastid label, the ones available on Zenodo at 10.5281/zenodo.18435290 (ref. ^55^) do not.

### Annotation consistency metrics

To assess the consistency of subcellular localization annotations between the HPA and UniProt databases, we evaluated the similarity of label sets for proteins annotated in both. We used several complementary metrics. ‘Equality’ measures the fraction of proteins with identical sets of labels. ‘Overlap’ measures the fraction of proteins with at least one shared label. Jaccard computes the average ratio of the intersection over the union of label sets. ‘Set Inclusion’ measures the fraction of proteins for which the label set from one database is a subset of the other. Formal definitions and equations for all metrics are provided in Supplementary Note.

### Confusion matrices

The confusion matrices in Fig. 1f,g as well as Extended Data Fig. 1d,e were constructed as follows. We maintained a 21 × 21 count matrix. For every protein, we looked at the overlap in their localization annotations. We looked at the common labels between the databases for Extended Data Fig. 1f,g and between the true and predicted labels for Extended Data Fig. 1d,e, and we added a one to the corresponding diagonal cell of the count matrix. For all labels not shared between the two annotations, we examined all possible pairs of disagreed-upon labels. For each pair of disagreed labels, we incremented the corresponding cell of the count matrix by one. If the labels from one annotation were a subset of the labels from the other annotation, we paired the disagreed labels with ‘-’ and incremented the count matrix accordingly. Then, the left matrix was computed by dividing the rows of the count matrix by the total count of the labels in HPA for Extended Data Fig. 1f,g and by the total count of ‘True’ labels in Extended Data Fig. 1d,e. Similarly, the right matrix was obtained by dividing the columns of the count matrix by the total count of the labels in UniProt for Extended Data Fig. 1f,g and by the total count of ‘Predicted’ labels for Extended Data Fig. 1d,e.

### Homology-aware partitioning and dataset construction

Following the approach of (cite LAProtT5), we used the ‘easy search’ command from mmseqs2 to align proteins in HOU to those in HPA and UniProt. If a protein pair exhibited ≥40% sequence identity for ≥80% of the longer protein, we ensured that both were either included in the HOU test set or excluded from it. Proteins that were not assigned to the HOU test set were used to construct training sets. We did not select a more stringent identity threshold to maintain a sufficient number of proteins in both the HOU test set and the training sets.

We constructed four training sets: (1) the HPA training was made from the remaining HPA proteins; (2) the UniProt training set was made from the remaining UniProt proteins; (3) the combined training set was made by taking the union of HPA and UniProt training sets and removing proteins that did not share the same level one localization annotations between the two sources; and (4) the combined (human) training set was derived by filtering the combined training set for human proteins.

Once all training sets were defined, we again used mmseqs2 to identify homologous proteins that must appear in separate training folds. We used single-linkage clustering to group sets of homologous proteins into non-overlapping clusters. This procedure ensures that any pair of homologous proteins appears in the same cluster. Finally, we used the StratifiedGroupKFold method from scikit-learn to merge protein clusters into five folds while preserving similar class frequencies across folds.

### Training existing models

All existing models were trained using fivefold cross-validation on our HPA-UniProt training set and evaluated on our HOU test set. For all models, the prediction threshold for each class was determined by maximizing the MCC on the validation set, as carried out by DeepLoc2 (ref. ^9^). Final predictions across folds were aggregated using majority voting, and final class probabilities were obtained by averaging probabilities across folds.

#### DeepLoc2

DeepLoc2 models are multilabel localization models built on top of PLM embeddings, specifically ProtT5 or ESM1b. The model uses a single attention head to aggregate residue embeddings across the sequence, followed by a classification head to generate multilabel localization predictions optimized with focal loss. To enhance biological interpretability, attention weights are guided toward annotated sorting signal regions using a Kullback–Leibler (KL) divergence loss term, and a Fourier-based regularization term encourages smoothness in the attention profile.

The original DeepLoc2 models were trained using sorting signal annotations curated from the literature for 1,768 proteins, only 791 of which were in our combined HPA-UniProt training set; these annotations were used during training, while the remaining proteins were included without sorting signal supervision. All hyperparameters followed the original work, with the coefficients for the Fourier and KL divergence losses set to 0.1. Protein sequences were truncated to the first and last 500 residues before PLM embedding. The learning rate was 1 × 10^−3^, and models were trained for up to 14 epochs with early stopping if validation performance did not improve for 5 epochs. The model with the best validation focal loss was saved.

#### MULocDeep

In MULocDeep, protein sequences are encoded using amino-acid descriptors and position-specific scoring matrices (PSSMs). Local hierarchical features are extracted via multiple convolutional and pooling layers, followed by BiLSTM layers for sequence context modeling. An attention mechanism aggregates information relevant to subcellular localization. MULocDeep outputs a probability matrix for hierarchical label prediction, allowing simultaneous prediction of both coarse- and fine-grained labels (for example, level 1 and level 3). For each coarse-grained class, the predicted probability is the maximum among its subclasses. Level 1 performance metrics are reported from the level 1–level 2 model, which achieved higher macro-averaged precision than the level 1–level 3 model.

PSSMs were generated using NCBI BLAST+: all SwissProt protein sequences were compiled into a BLAST database with makeblastdb, and PSSMs were constructed using the psi-blast command^56^. Key hyperparameters matched the original implementation: 64 convolutional filters, 128 LSTM units, a learning rate of 1 × 10^−3^, batch size of 128, and dropout rate of 0.5. Models were trained for up to 30 epochs with early stopping based on validation loss.

#### LAProtT5

LAProtT5 is a single-label localization predictor leveraging ProtT5 embeddings. It applies a one-dimensional convolutional layer to capture local sequence patterns, followed by a specialized light attention module to aggregate information across the sequence and a dense prediction head. Rather than modifying the original LAProtT5 codebase, we evaluated the architecture using a specific configuration from our hyperparameter sweep. Key hyperparameters were retained from the original model: convolutional kernel size of 9, convolutional dropout of 0.25, classification head dropout of 0.25 and learning rate of 1 × 10^−3^. Our implementation differs from the original in several respects: we use binary cross-entropy, rather than cross-entropy, loss to support multilabel prediction, train for up to 100 epochs, instead of 30, and replace the original single-layer, 32-dimensional classification head with a three-layer head with a 512-dimensional hidden layer.

#### Random baseline

The random baseline was defined as a Bernoulli model, in which each class label is independently predicted with probability equal to the frequency in the HPA-UniProt combined training set. All performance metrics were analytically estimated using class frequencies in the training and test sets.

### Systematic evaluation of PLM and aggregation method combinations

We conducted a comprehensive ablation study to identify optimal configurations for evaluating PLMs in the localization task. We used four different PLMs, ESM2 (esm2_t36_3B), ESM3-small open, ProtT5 and ProtBert. We also evaluated different aggregation methods, including mean, max, light attention and multihead attention pooling. The experimental sweep included the following hyperparameters: sequence length (512, 1,024 or 2,048), classifier dropout rate after aggregation (0, 0.25 or 0.5) and choice of classification loss (cross-entropy or focal loss^26^). Finally, the training datasets were selected from the HPA dataset, UniProt dataset, the combined HPA and UniProt dataset, and a subset comprising only human data from both HPA and UniProt. This grid-search approach allowed us to systematically analyze the influence of each parameter on the model’s performance in the protein localization task.

**Extended Data Fig. 7:**
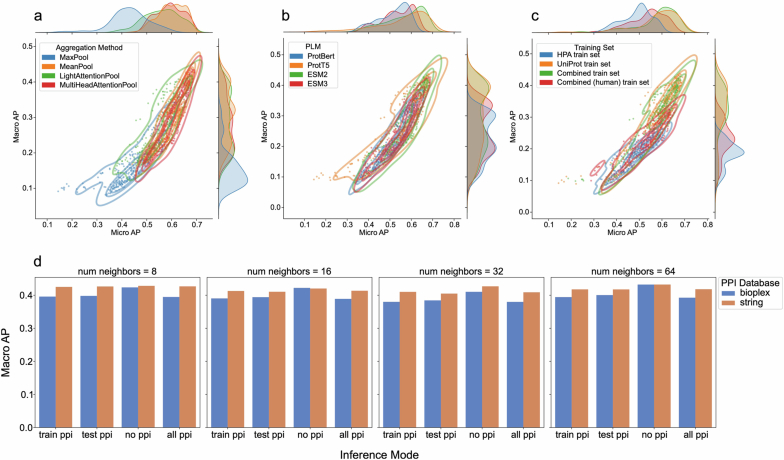
Impact of hyperparameters on model performance. Density plot of results from hyperparameter sweep for systematic evaluation of PLM and aggregation strategy combinations. Marco and micro AP for across all model combinations colored by **a**, aggregation strategy, **b**, PLM, **c**, training set. **d**, Bar plot showing performance of PPI-integrated localization predictors when trained with various number of neighbors during training, using different PPI datasets and across various inference modes.

We did not notice consistent performance trends across sequence, length, dropout or loss. Performance across PLM, aggregation strategy and dataset are visualized in Extended Data Fig. 7a–c. We observed comparable performance between models trained on the UniProt and Combine training sets, and chose to report performance of models trained on the latter as it is larger.

All models in the sweep were trained with an initial learning rate of 1 × 10^−3^, which was progressively reduced by a factor of 0.5 if no improvement was observed for four consecutive epochs. The training was performed for a total of 100 epochs, with early stopping criteria applied if no improvement in validation set performance was observed for 20 consecutive epochs. We chose macro AP as the evaluation metric to monitor model performance during training, ensuring a balanced assessment across different classes. As with existing models, class prediction thresholds were set by maximizing MCC on the validation set. Final predictions were aggregated across folds using majority voting, and class probabilities were averaged across folds.

### Evaluation metrics

We evaluated all models using a comprehensive set of metrics consistent with standard multilabel classification practices. Per-class performance was assessed using MCC, F1 score, precision, Jaccard index, recall and accuracy. To capture ranking quality, we additionally reported AP and receiver operating characteristic–area under the curve. Overall performance was summarized using both macro- and micro-averages of per-class metrics, as well as per-sample label ranking metrics, including coverage error and mean label ranking AP. Formal definitions, equations and innate biases for all metrics are described in Supplementary Note.

### Stratification by protein property

We used UniProt annotations to classify proteins as membrane-associated and to identify the presence of signal or transit peptides. UniProt was also used to extract structural features, including the number and length of α helices, β strands and turns. For physicochemical properties, amino acids were grouped as follows: F, I, W, L, V and M as hydrophobic; R, K, N, E, P and D as hydrophilic; R, H and K as positively charged; and D and E as negatively charged. Proteins were labeled as intrinsically disordered if they were listed in DisProt (a manually curated database of intrinsically disordered proteins^57^). For continuous properties, we binned values such that the smallest bin contained at least 10% of proteins in the HOU test set.

### Attention peaks and motif discovery

We examined whether attention profiles from ProtT5-MHA aligned with known protein motifs and subcellular localization signals. For each protein in the HOU test set we extracted the attention profile. The attention values for each profile were normalized by *z*-score standardization. Sequences were zero-padded up to length 1,024 for alignment. Peaks were identified using a moving-average smoothing window of five residues with a decision threshold of 0.5. We retained peaks with a minimum height of 1.0 and whose length exceeded five residues. To ensure enough contextual information, each detected peak was symmetrically expanded or trimmed to a fixed 50-residue window centered at the peak apex with boundary-aware handling at sequence termini. Overlapping attention peak windows were combined.

We compared attention peak segments to known sequence motifs from two sources. Functional and family domain motifs for HOU proteins were obtained by scanning sequences against PROSITE^28^ using ScanProsite service^55,58,59^. Sorting signals were obtained from DeepLoc2, which curated a set of proteins with known sorting signals from the literature. Intersecting this set with our HOU test set and filtering for identical canonical isoforms yielded 184 proteins with annotated sorting signals. We considered an attention peak and motif to overlap if 50% of the motif was covered by the attention peak segment.

To search for novel sorting signals, we isolated proteins for which ProtT5-MHA predicted at least one correct localization. We further filtered to only include proteins for which none of the attention peaks overlap more than 10% with PROSITE motifs or annotated sorting signals. This ensures that correct predictions are not driven by other known motifs. With this set of proteins we clustered motifs per localization classes using BLOSUM62 to measure sequence similarity on short motifs. We found 17 total clusters of motifs with five or more proteins. Four of them corresponded to known nuclear localizing signals, such as KKQ or KKXK, for proteins not in our annotated set from DeepLoc2, and so we eliminated them. In Supplementary Table 15 we show the nine motifs associated with three or more species.

### PPI-integrated model training

To integrate the PPI information into the language models for localization prediction, we employed GraphSAGE^32^ graph neural network layers after the aggregator layer in the original architecture. During training, we fed the subgraph of the PPIs with a batch size of 8 and performed a hyperparameter sweep on the number of neighbors to evaluate its impact on performance. We tested using 8, 16, 32 or 64 neighboors. During inference, we fixed the number of neighbors to 64 and tested the models with different interaction graphs (only interactions present in the training set, only interactions present in the test set, all the interactions present in the database and no interactions).

Experiments revealed higher performance when training on STRING data rather than BioPlex data and that 64 neighbors resulted in the best performance for level 1 annotations (Extended Data Fig. 7d).

### Reporting summary

Further information on research design is available in the Nature Portfolio Reporting Summary linked to this article.

## Online content

Any methods, additional references, Nature Portfolio reporting summaries, source data, extended data, supplementary information, acknowledgements, peer review information; details of author contributions and competing interests; and statements of data and code availability are available at 10.1038/s41592-026-03142-6.

## Supplementary information

Supplementary Information Supplementary Notes and Tables 1–17.

Reporting Summary

Supplementary Data 1 List of proteins with no common localization annotation.

## Data Availability

All raw data used to construct our datasets and for all downstream analysis can be found on Zenodo at https://zenodo.org/records/18275384 (ref. ^59^). Our proposed training sets, HOU localization test set and hierarchical label set, and mapping can be found on Zenodo at 10.5281/zenodo.18435290 (ref. ^55^).

## Code availability

Code for creating datasets as well as training and evaluating models can be found on GitHub at https://github.com/CellProfiling/seq2loc_benchmark/tree/main.
