# Supplementary material for: A comprehensive benchmark of sequence-based subcellular localization predictors for human proteins
Source: Nat Methods. 2026 Jul 6;23(7):1458–69. doi: 10.1038/s41592-026-03142-6 (PMC13345906; doi:10.1038/s41592-026-03142-6)
Supplement: Supplementary file 1 — Supplementary Notes and Tables 1–17. [file 41592_2026_3142_MOESM1_ESM.pdf]

nature methods

Supplementary information

https://doi.org/10.1038/s41592-026-03142-6

A comprehensive benchmark of sequence-based subcellular localization predictors for human proteins

In the format provided by the authors and unedited

Contents

1 Supplementary Note **2**

1.1 Annotation Consistency Metrics . . . 2

1.2 Evaluation Metrics . . . 2

2 Supplementary Tables **4**

SI Table 1: Hierarchical Label Set . . . 4

SI Table 2: Frequencies of level 1 labels . . . 5

SI Table 3: Frequencies of level 2 labels . . . 5

SI Table 4: Frequencies of level 3 labels . . . 5

SI Table 5: Tool usability . . . 6

SI Table 6: Model performance by averaged metrics . . . 6

SI Table 7: Baseline model performance per class for level 1 labels . . . 7

SI Table 8: Baseline model performance per class for level 2 labels . . . 8

SI Table 9: Baseline model performance per class for level 3 labels . . . 9

SI Table 10: Class size influence on averaged metrics . . . 10

SI Table 11: Optimal parameters for PLM-based models . . . 10

SI Table 12: PLM Benchmark model performances per class for level 1 labels . . . 11

SI Table 13: PLM Benchmark model performances per class for level 2 labels . . . 12

SI Table 14: PLM Benchmark model performances per class for level 2 labels . . . 13

SI Table 16: Fuzzy sequence motifs from attention peaks . . . 14

SI Table 16: Model performance with PPI-integration . . . 15

SI Table 17: Influence of PPI-integration per class . . . 15

1 Supplementary Note

1.1 Annotation Consistency Metrics

Let H_i and S_i denote the sets of subcellular localization labels for the ith protein in the Human Protein Atlas (HPA) and SwissProt (UniProt), respectively. Let N be the number of proteins with annotations in both databases. We use the following four metrics to quantify consistency.

- **Equality**: Fraction of proteins for which the label sets match exactly.

$$\text{Equality} = \frac{1}{N}\sum_{i=1}^{N} \nVdash (H_i = S_i)$$

- **Overlap**: Fraction of proteins with at least one shared label.

$$\text{Overlap} = \frac{1}{N}\sum_{i=1}^{N} \nVdash (H_i \cap S_i \neq \emptyset)$$

- **Jaccard Index**: Average Jaccard similarity between label sets.

$$\text{Jaccard} = \frac{1}{N}\sum_{i=1}^{N} \frac{|H_i \cap S_i|}{|H_i \cup S_i|}$$

- **Set Inclusion**: Fraction of proteins where one label set is a subset of the other.

$$\text{Set-Inclusion} = \frac{1}{N}\sum_{i=1}^{N} \mathbf{1}(H_i \subseteq S_i \text{ or } S_i \subseteq H_i)$$

where $\mathbf{1}(\cdot)$ denotes the indicator function, which evaluates to 1 if the condition is true and 0 otherwise.

1.2 Evaluation Metrics

- **Matthews Correlation Coefficient (MCC)** (per class):

$$MCC = \frac{(TP \cdot TN) - (FP \cdot FN)}{\sqrt{(TP + FP)(TP + FN)(TN + FP)(TN + FN)}}$$

  Biases and Advantages: Equally penalizes false positives and false negatives. Incorporates all four confusion matrix components. Robust to class imbalance. Less intuitive than simpler metrics.

- **Accuracy** (per class):

$$ACC = \frac{TP + TN}{TP + TN + FP + FN}$$

  Biases and Advantages: Can be misleading for rare classes, as a model predicting only negatives can achieve high accuracy.

- **Precision** (per class):

$$Precision = \frac{TP}{TP + FP}$$

  Biases and Advantages: High when the model avoids false positives. Can be misleading if the model rarely predicts the class.

- **Recall** (per class):

$$Recall = \frac{TP}{TP + FN}$$

Biases and Advantages: High when the model captures all positives. Can be misleading if the model over-predicts the class.

- **F1 Score** (per class):

$$F1 = \frac{2 \cdot Precision \cdot Recall}{Precision + Recall} = \frac{2 \cdot TP}{2 \cdot TP + FP + FN}$$

Biases and Advantages: Equally prioritizes precision and recall. Does not reward true negatives, leading to lower scores for rare classes.

- **Average Precision (AP)** (per class): Let P_n be the precision at the nth threshold. Let R_n be the recall at the nth threshold. Thresholds are determined by ranking predictions by probability in decreasing order. The recall and precision at the nth threshold take into account only predictions with probabilities ranked at or above the nth threshold. AP is the area under the precision-recall curve.

$$AP = \sum_n (R_n - R_{n-1}) \cdot P_n$$

Biases and Advantages: Robust to class imbalance. Threshold independent.

- **ROC-AUC** (perclass): Let TPR_n and FPR_n be the true positive rate and false positive rate at the nth threshold, respectively. $TPR = \frac{TP}{TP+FN}$ $FPR = \frac{FP}{FP+TN}$. The ROC-AUC is the area under the receiver operating characteristic curve which the TPR against the FPR.

$$ROC\text{-}AUC = \sum_n (FPR_n - FPR_{n-1}) \cdot \frac{TPR_n + TPR_{n-1}}{2}$$

Biases and Advantages: Threshold independent. Overly optimistic for rare classes because FPR remain low by default.

- **Jaccard Index** (per class):

$$Jaccard = \frac{|\text{Predicted} \cap \text{True}|}{|\text{Predicted} \cup \text{True}|} = \frac{TP}{TP + FP + FN}$$

Biases and Advantages: Strict measure that equally penalizes false positives and false negatives. Does not reward true negatives. Thus it may underestimate performance for rare classes

- **Coverage Error**: Let each test sample x_i have a set of true labels $Y_i \subset \{1, 2, ..k\}$, where k is the total number of classes. Let $f_i = [f_{i1}, f_{i2}, ...f_{ik}]$ be the predicted probabilities of each class. Le N be the number of samples. The coverage is the largest rank of any true positive class in f_i, and the coverage error is the average coverage error overall samples.

$$\text{Coverage Error} = \frac{1}{N} \sum_{i=1}^{N} \left(\max_{y \in Y_i} \text{rank}_i(y) + 1 \right)$$

Biases and Advantages: Useful for evaluating ranking quality in multilabel tasks. Less interpretable as a classification metric.

- **Mean Label Ranking Average Precision (MLRAP)**: For sample x_i, with true labels Y_i and predicted scores f_i, define R_{ij} as the set of labels ranked above label j. The LRAP score for a sample is:

$$LRAP_i = \frac{1}{|Y_i|} \sum_{j \in Y_i} \frac{|R_{ij} \cap Y_i|}{|R_{ij}|}$$

The final score is:

$$MLRAP = \frac{1}{N} \sum_{i=1}^{N} LRAP_i$$

Biases and Advantages: Useful for evaluating ranking quality in multilabel tasks. Less interpretable as a classification metric.

2 Supplementary Tables

**Level** 1 (22 classes)	**Level 2** (10 classes)	**Level 3** (8 classes)	**DeepLoc** (10 classes)
Cytoskeleton	Cytoskeleton	Cytoskeleton	
Actin-filaments			
Intermediate-filaments			
Centrosome			
Microtubules			
Plasma-membrane	Plasma-membrane	Plasma-membrane	Plasma-membrane
Cytosol	Cytosol	Cytosol	Cytoplasm
Endoplasmic-reticulum	Endoplasmic-reticulum	Endomembrane-system	Endoplasmic-reticulum
Golgi-apparatus	Golgi-apparatus		Golgi-apparatus
Vesicles	Vesicles		
Endosomes			
Lysosomes			Lysosomes/Vacuole
Peroxisomes			Peroxisomes
Lipid-droplets			
Mitochondria	Mitochondria	Mitochondria	Mitochondria
Nucleoplasm	Nucleus	Nucleus	Nucleus
Nuclear-bodies			
Nuclear-membrane			
Nuclear-speckles			
Nucleoli	Nucleoli	Nucleoli	
Nucleoli-fibrillar-center			
Plastid	Plastid	Plastid	Plastid
			Extracellular

SI Table 1: The hierarchical label set constructed for this study, compared against the set of compartment labels defined by the initial DeepLoc publication.

Locations	HPA	OpenCell	UniProt	HOU test set	HPA train set	UniProt train set	Combined trainset	Combined (human) trainset
**cytoskeleton**	0 (0.00%)	29 (2.22%)	1025 (3.39%)	7 (0.18%)	0 (0.00%)	714 (3.42%)	699 (2.64%)	91 (1.06%)
**actin-filaments**	336 (3.15%)	0 (0.00%)	761 (2.52%)	29 (0.76%)	268 (3.38%)	427 (2.04%)	603 (2.28%)	257 (2.99%)
**intermediate-filaments**	147 (1.27%)	0 (0.00%)	0 (0.00%)	6 (0.16%)	121 (1.53%)	0 (0.00%)	87 (0.33%)	87 (1.01%)
**centrosome**	572 (4.30%)	50 (3.83%)	575 (1.90%)	125 (3.28%)	360 (4.54%)	258 (1.23%)	525 (1.99%)	399 (4.64%)
**microtubules**	254 (2.19%)	0 (0.00%)	990 (3.27%)	39 (1.02%)	177 (2.23%)	538 (2.57%)	650 (2.46%)	278 (3.23%)
**plasma-membrane**	2181 (18.8%)	135 (10.3%)	6151 (20.3%)	407 (10.7%)	1496 (18.9%)	4314 (20.6%)	5240 (19.8%)	1998 (23.3%)
**cytosol**	4829 (41.6%)	483 (37.0%)	11863 (39.2%)	1291 (33.9%)	3158 (39.8%)	7495 (35.9%)	9720 (36.8%)	3325 (38.7%)
**endoplasmic-reticulum**	480 (4.14%)	95 (7.28%)	2453 (8.11%)	166 (4.35%)	276 (3.48%)	1736 (8.30%)	1863 (7.05%)	496 (5.77%)
**golgi-apparatus**	1107 (9.54%)	64 (4.90%)	1489 (4.92%)	145 (3.80%)	802 (10.1%)	972 (4.65%)	1559 (5.90%)	793 (9.23%)
**vesicles**	2072 (17.9%)	262 (20.1%)	1018 (3.37%)	165 (4.33%)	1543 (19.5%)	694 (3.32%)	1873 (7.09%)	1280 (14.9%)
**endosomes**	16 (0.14%)	0 (0.00%)	981 (3.24%)	117 (3.07%)	7 (0.09%)	534 (2.55%)	494 (1.87%)	113 (1.31%)
**lysosomes**	19 (0.16%)	0 (0.00%)	605 (2.00%)	119 (3.12%)	9 (0.11%)	265 (1.27%)	239 (0.90%)	96 (1.12%)
**peroxisomes**	23 (0.20%)	0 (0.00%)	413 (1.37%)	11 (0.29%)	12 (0.15%)	333 (1.59%)	325 (1.23%)	28 (0.33%)
**lipid-droplets**	37 (0.32%)	0 (0.00%)	180 (0.60%)	24 (0.63%)	21 (0.26%)	121 (0.58%)	138 (0.52%)	31 (0.36%)
**mitochondria**	1029 (8.86%)	11 (0.84%)	3132 (10.4%)	335 (8.78%)	620 (7.82%)	2173 (10.4%)	2627 (9.94%)	749 (8.72%)
**nucleoplasm**	5807 (50.0%)	425 (32.6%)	10628 (35.1%)	1618 (42.4%)	3793 (47.9%)	6699 (32.0%)	9575 (36.2%)	3506 (40.8%)
**nuclear-bodies**	954 (8.22%)	77 (5.90%)	130 (0.43%)	86 (2.25%)	608 (7.67%)	46 (0.22%)	510 (1.93%)	477 (5.55%)
**nuclear-membrane**	282 (2.43%)	32 (2.45%)	540 (1.79%)	48 (1.26%)	189 (2.38%)	377 (1.80%)	507 (1.92%)	180 (2.09%)
**nuclear-speckles**	485 (4.18%)	0 (0.00%)	278 (0.92%)	33 (0.87%)	356 (4.49%)	115 (0.55%)	353 (1.34%)	262 (3.05%)
**nucleoli**	1089 (9.38%)	76 (5.82%)	955 (3.16%)	170 (4.46%)	687 (8.67%)	521 (2.49%)	1040 (3.93%)	582 (6.77%)
**nucleoli-fibrillar-center**	349 (3.01%)	22 (1.69%)	1 (0.00%)	22 (0.58%)	241 (3.04%)	1 (0.00%)	180 (0.68%)	180 (2.09%)
**plastid**	0 (0.00%)	0 (0.00%)	1160 (3.83%)	0 (0.00%)	0 (0.0%)	1142 (5.46%)	1142 (4.32%)	0 (0.00%)
**Total**	**11,608**	**1,305**	**32,843**	**3,814**	**7,927**	**20,906**	**26,436**	**8,594**

SI Table 2: Frequencies of level 1 labels in databases and constructed datasets.

Locations	HPA	OpenCell	UniProt	HOU test set	HPA train set	UniProt train set	Combined trainset	Combined (human) trainset
**cytoskeleton**	1284 (11.1%)	79 (6.05%)	2737 (9.05%)	195 (5.11%)	890 (11.2%)	2154 (18.6%)	1029 (8.86%)	6451 (55.6%)
**plasma-membrane**	2181 (18.8%)	135 (10.3%)	6151 (20.3%)	407 (10.7%)	1496 (18.8%)	427 (2.04%)	603 (2.28%)	257 (2.99%)
**cytosol**	4829 (41.6%)	483 (37.0%)	11863 (39.2%)	1291 (33.9%)	3158 (39.8%)	0 (0.00%)	87 (0.33%)	87 (1.01%)
**endoplasmic-reticulum**	480 (4.14%)	95 (7.28%)	2453 (8.11%)	166 (4.35%)	276 (3.48%)	258 (1.23%)	525 (1.99%)	399 (4.64%)
**golgi-apparatus**	1107 (9.54%)	64 (4.90%)	1489 (4.92%)	145 (3.80%)	802 (10.1%)	538 (2.57%)	650 (2.46%)	278 (3.23%)
**vesicles**	2154 (18.6%)	262 (20.1%)	2971 (9.82%)	384 (10.1%)	1586 (20.0%)	4314 (20.6%)	5240 (19.8%)	1998 (23.3%)
**mitochondria**	1029 (8.86%)	11 (0.84%)	3132 (10.4%)	335 (8.78%)	620 (7.82%)	7495 (35.9%)	9720 (36.8%)	3325 (38.7%)
**nucleus**	6451 (55.6%)	479 (36.7%)	11037 (36.5%)	1709 (44.8%)	4271 (53.9%)	1736 (8.30%)	1863 (7.05%)	496 (5.77%)
**nucleoli**	1292 (12.0%)	98 (7.51%)	955 (3.16%)	196 (5.14%)	892 (11.3%)	972 (4.65%)	1559 (5.90%)	793 (9.23%)
**plastid**	0 (0.00%)	0 (0.00%)	1160 (3.83%)	0 (0.00%)	0 (0.0%)	1142 (5.46%)	1142 (4.32%)	0 (0.00%)
**Total**	**11,608**	**1,305**	**32,843**	**3,814**	**7,927**	**20,906**	**26,436**	**8,594**

SI Table 3: Frequencies of level 2 labels in databases and constructed datasets.

Locations	HPA	OpenCell	UniProt	HOU test set	HPA train set	UniProt train set	Combined trainset	Combined (human) trainset
**cytoskeleton**	1284 (11.1%)	79 (6.05%)	2737 (9.05%)	195 (5.11%)	890 (11.2%)	2154 (18.6%)	1029 (8.86%)	6451 (55.6%)
**plasma-membrane**	2181 (18.8%)	135 (10.3%)	6151 (20.3%)	407 (10.7%)	1496 (18.8%)	427 (2.04%)	603 (2.28%)	257 (2.99%)
**cytosol**	4829 (41.6%)	483 (37.0%)	11863 (39.2%)	1291 (33.9%)	3158 (39.8%)	0 (0.00%)	87 (0.33%)	87 (1.01%)
**endomembrane-system**	3374 (29.1%)	376 (28.8%)	6062 (20.0%)	669 (17.5%)	2413 (30.0%)	4065 (19.4%)	5737 (21.7%)	2496 (29.0%)
**mitochondria**	1029 (8.86%)	11 (0.84%)	3132 (10.4%)	335 (8.78%)	620 (7.82%)	7495 (35.9%)	9720 (36.8%)	3325 (38.7%)
**nucleus**	6451 (55.6%)	479 (36.7%)	11037 (36.5%)	1709 (44.8%)	4271 (53.9%)	1736 (8.30%)	1863 (7.05%)	496 (5.77%)
**nucleoli**	1292 (12.0%)	98 (7.51%)	955 (3.16%)	196 (5.14%)	892 (11.3%)	972 (4.65%)	1559 (5.90%)	793 (9.23%)
**plastid**	0 (0.00%)	0 (0.00%)	1160 (3.83%)	0 (0.00%)	0 (0.0%)	1142 (5.46%)	1142 (4.32%)	0 (0.00%)
**Total**	**11,608**	**1,305**	**32,843**	**3,814**	**7,927**	**20,906**	**26,436**	**8,594**

SI Table 4: Frequencies of level 3 labels in databases and constructed datasets.

Name	Method	Code Available	Weights Available	Documentation	Runnable	Year Published
YLoc+	Webserver	Github Repo	-	Limited	No - webserver down	2010
DeepLoc1	Model	Github Repo	No	Comprehensive	No - outdated libraries	2017
Fuel-mLoc	Webserver	No	-	Limited	No - webserver down	2017
LAProtT5	Model	Github Repo	No	Comprehensive	Yes	2021
MuLocDeep	Model	Github Repo	Yes, Github	Comprehensive	Yes	2021
DeepLoc2	Model	Github Repo	No	Comprehensive	Yes	2022
ML-FGAT	Model	Github Repo	No	Limited	No	2024
ESM1	PLM	Github Repo	Yes, Hugging Face	Comprehensive	Yes	2021
ProtBert	PLM	Github Repo	Yes, Hugging Face	Comprehensive	Yes	2022
ProtT5	PLM	Github Repo	Yes, Hugging Face	Comprehensive	Yes	2022
ESM2	PLM	Github Repo	Yes, Hugging Face	Comprehensive	Yes	2023
ESM3-open	PLM	Github Repo	Yes, Hugging Face	Comprehensive	Yes	2025

SI Table 5: Usability of modern protein sequence to localization tools and protein language models.

**Model**	**Accuracy**			**Macro AP**			**Micro AP**			**Macro F1**			**Micro F1**			**Num Labels**		
	1	2	3	1	2	3	1	2	3	1	2	3	1	2	3	1	2	3
Random	0.050	0.068	0.071	-	-	-	-	-	-	0.065	0.132	0.163	0.221	0.257	0.275	**1.613**	1.468	1.443
MuLocDeep	0.227	0.354	0.391	0.290	0.552	0.634	*0.590*	0.672	0.687	0.304	0.550	0.601	0.460	0.621	0.640	2.341	1.489	1.474
DeepLoc2-ProtT5	0.078	0.334	0.448	0.332	0.584	0.669	0.401	0.672	0.718	0.328	0.569	0.642	0.416	0.631	0.688	3.514	1.755	1.397
DeepLoc2-ESM1	0.109	0.356	0.453	0.324	0.590	0.662	0.423	0.671	0.703	0.339	0.581	0.642	0.422	0.631	0.686	3.300	1.612	1.404
LAProtT5	*0.238*	*0.407*	*0.512*	*0.371*	*0.614*	*0.682*	0.582	*0.735*	*0.755*	*0.383*	*0.584*	*0.653*	*0.521*	*0.654*	*0.705*	1.910	*1.392*	*1.225*
ESM2	0.232	0.458	0.518	0.422	**0.651**	**0.717**	0.670	0.746	0.780	**0.416**	0.613	**0.674**	0.537	0.647	0.713	2.109	**1.255**	1.218
ESM3	0.245	0.402	0.476	0.372	0.587	0.667	0.631	0.708	0.740	0.368	0.553	0.644	0.529	0.646	0.689	2.007	1.329	**1.261**
ProtT5	**0.272**	**0.476**	**0.537**	**0.428**	0.649	0.709	**0.684**	**0.748**	**0.787**	0.403	**0.622**	0.669	**0.585**	**0.690**	**0.718**	*1.852*	1.314	1.193
ProtBert	0.122	0.281	0.431	0.336	0.565	0.649	0.606	0.681	0.716	0.303	0.548	0.619	0.413	0.596	0.668	3.058	1.727	1.327

**Model**	**Macro Jaccard**			**Micro Jaccard**			**Macro AUC**			**Micro AUC**			**MLRAP**			**Coverage Error**		
	1	2	3	1	2	3	1	2	3	1	2	3	1	2	3	1	2	3
Random	0.037	0.076	0.096	0.124	0.147	0.160	-	-	-	-	-	-	-	-	-	-	-	-
MuLocDeep	0.206	0.394	0.442	*0.299*	0.450	**0.640**	0.835	0.866	0.874	0.905	0.906	0.898	*0.747*	0.783	0.803	3.698	2.179	1.984
DeepLoc2-ProtT5	0.223	0.419	0.486	0.262	0.461	0.524	0.850	0.897	*0.903*	0.875	0.908	0.905	0.634	0.791	0820	4.267	2.152	1.942
DeepLoc2-ESM1	0.232	0.427	0.487	0.268	0.461	0.522	0.845	0.891	0.893	0.874	0.904	0.897	0.637	0.786	0.811	4.271	2.189	1.974
LAProtT5	*0.266*	*0.436*	*0.500*	0.352	*0.486*	0.545	*0.867*	*0.899*	0.902	*0.921*	*0.928*	*0.926*	0.742	*0.818*	*0.846*	*3.143*	*2.006*	*1.792*
ESM2	0.294	0.460	0.520	0.367	0.508	0.554	0.873	**0.970**	**0.912**	0.939	0.933	0.933	0.803	0.826	0.854	2.842	1.967	1.769
ESM3	0.250	0.401	0.486	0.360	0.477	0.525	0.850	0.889	0.893	0.928	0.922	0.918	0.782	0.805	0.831	3.042	2.028	1.885
ProtT5	0.281	0.470	0.516	**0.414**	**0.527**	*0.560*	**0.882**	0.908	0.910	**0.944**	**0.935**	**0.935**	**0.809**	**0.834**	**0.861**	**2.726**	**1.930**	**1.743**
ProtBert	0.201	0.399	0.462	0.260	0.425	0.502	0.835	0.871	0.878	0.925	0.910	0.909	0.760	0.787	0.816	3.131	2.132	1.910

SI Table 6: Averaged metrics for existing baseline models (top) and best performing systematic-PLM models (bottom). Results are shown for models trained at each level of hierarchical label set. The best results for existing baseline models systematic-PLM models are emphasized. The best result is bolded and the second-best result is italicized.

	Random							MuLocDeep							DeepLoc2-ProtT5							DeepLoc2-ESM1							LAProtT5						
	MCC	ACC	Recall	Prec	F1	Jacc	AUC	MCC	ACC	Recall	Prec	F1	Jacc	AUC	MCC	ACC	Recall	Prec	F1	Jacc	AUC	MCC	ACC	Recall	Prec	F1	Jacc	AUC	MCC	ACC	Recall	Prec	F1	Jacc	AUC
cytoskeleton	0.000	0.872	0.086	0.050	0.064	0.033	-	0.359	0.906	0.573	0.284	0.379	0.234	0.877	0.364	0.842	0.807	0.215	0.340	0.205	0.898	0.327	0.832	0.755	0.196	0.311	0.184	0.893	0.411	0.938	0.490	0.402	0.441	0.283	0.895
actin-filaments	0.000	0.970	0.023	0.008	0.011	0.006	-	0.121	0.801	0.759	0.028	0.055	0.028	0.856	0.198	0.924	0.690	0.066	0.121	0.065	0.909	0.170	0.948	0.483	0.071	0.124	0.066	0.885	0.174	0.966	0.379	0.091	0.147	0.079	0.928
intermediate-filaments	0.000	0.995	0.003	0.002	0.002	0.001	-	0.046	0.696	0.833	0.004	0.009	0.004	0.782	-0.001	0.998	0.000	0.000	0.000	0.000	0.739	-0.001	0.998	0.000	0.000	0.000	0.000	0.830	0.029	0.795	0.500	0.004	0.008	0.004	0.859
centrosome	0.000	0.949	0.020	0.033	0.025	0.013	-	0.188	0.960	0.152	0.284	0.198	0.110	0.874	0.228	0.823	0.672	0.117	0.199	0.111	0.863	0.234	0.856	0.608	0.131	0.216	0.121	0.865	0.377	0.944	0.536	0.303	0.387	0.240	0.880
microtubules	0.000	0.966	0.025	0.010	0.014	0.007	-	0.159	0.931	0.462	0.069	0.121	0.064	0.868	0.124	0.881	0.513	0.044	0.081	0.042	0.839	0.160	0.896	0.590	0.057	0.104	0.055	0.862	0.236	0.970	0.410	0.151	0.221	0.124	0.889
plasma-membrane	0.000	0.737	0.198	0.107	0.139	0.075	-	0.574	0.921	0.595	0.642	0.617	0.446	0.904	0.604	0.917	0.713	0.593	0.647	0.479	0.931	0.578	0.908	0.715	0.552	0.623	0.453	0.920	0.633	0.933	0.634	0.709	0.669	0.503	0.927
cytosol	0.000	0.543	0.368	0.338	0.352	0.214	-	0.408	0.676	0.833	0.513	0.635	0.466	0.786	0.476	0.723	0.839	0.561	0.672	0.507	0.805	0.456	0.707	0.844	0.544	0.661	0.494	0.791	0.443	0.723	0.763	0.568	0.651	0.483	0.809
endoplasmic-reticulum	0.000	0.892	0.070	0.044	0.054	0.028	-	0.567	0.959	0.663	0.521	0.584	0.412	0.931	0.501	0.937	0.729	0.384	0.503	0.336	0.949	0.535	0.952	0.669	0.466	0.550	0.379	0.939	0.620	0.972	0.548	0.734	0.628	0.457	0.951
golgi-apparatus	0.000	0.907	0.059	0.038	0.046	0.024	-	0.355	0.964	0.248	0.554	0.343	0.207	0.839	0.252	0.953	0.228	0.333	0.270	0.156	0.872	0.322	0.953	0.331	0.364	0.347	0.210	0.888	0.286	0.965	0.117	0.739	0.202	0.113	0.856
vesicles	0.000	0.810	0.113	0.100	0.106	0.056	-	0.294	0.870	0.377	0.357	0.367	0.225	0.756	0.258	0.527	0.945	0.168	0.286	0.167	0.825	0.303	0.728	0.746	0.232	0.354	0.215	0.814	0.326	0.838	0.537	0.317	0.398	0.249	0.811
endosomes	0.000	0.952	0.019	0.031	0.023	0.012	-	0.136	0.951	0.154	0.168	0.161	0.087	0.791	0.167	0.945	0.214	0.177	0.194	0.107	0.851	0.125	0.956	0.120	0.179	0.144	0.077	0.854	0.153	0.957	0.145	0.210	0.172	0.094	0.847
lysosomes	0.000	0.960	0.009	0.031	0.014	0.007	-	0.163	0.950	0.185	0.193	0.189	0.104	0.805	0.287	0.921	0.513	0.201	0.289	0.169	0.885	0.355	0.937	0.563	0.261	0.356	0.217	0.885	0.219	0.953	0.244	0.244	0.244	0.139	0.842
peroxisomes	0.000	0.985	0.012	0.003	0.005	0.002	-	0.212	0.997	0.091	0.500	0.154	0.083	0.883	0.184	0.988	0.364	0.098	0.154	0.083	0.959	0.104	0.996	0.091	0.125	0.105	0.056	0.958	0.602	0.998	0.545	0.667	0.600	0.429	0.991
lipid-droplets	0.000	0.989	0.005	0.006	0.006	0.003	-	0.150	0.981	0.250	0.100	0.143	0.077	0.872	0.393	0.993	0.333	0.471	0.390	0.242	0.967	0.416	0.991	0.500	0.353	0.414	0.261	0.942	0.338	0.994	0.208	0.556	0.303	0.179	0.924
mitochondria	0.000	0.830	0.099	0.088	0.093	0.049	-	0.764	0.964	0.731	0.839	0.781	0.641	0.950	0.814	0.971	0.779	0.882	0.827	0.705	0.965	0.799	0.970	0.734	0.904	0.811	0.681	0.961	0.844	0.976	0.800	0.918	0.855	0.747	0.949
nucleoplasm	0.000	0.521	0.362	0.424	0.391	0.243	-	0.523	0.769	0.680	0.752	0.714	0.555	0.853	0.618	0.815	0.724	0.818	0.768	0.624	0.889	0.598	0.805	0.742	0.786	0.763	0.617	0.882	0.608	0.809	0.672	0.846	0.749	0.599	0.878
nuclear-bodies	0.000	0.959	0.016	0.023	0.021	0.011	-	0.049	0.894	0.186	0.045	0.073	0.038	0.731	0.062	0.599	0.605	0.034	0.064	0.033	0.668	0.036	0.519	0.605	0.028	0.054	0.028	0.622	0.109	0.777	0.523	0.053	0.096	0.050	0.766
nuclear-membrane	0.000	0.969	0.019	0.013	0.015	0.008	-	0.256	0.988	0.083	0.800	0.151	0.082	0.737	0.356	0.988	0.250	0.522	0.338	0.203	0.839	0.325	0.987	0.229	0.478	0.310	0.183	0.827	0.437	0.990	0.229	0.846	0.361	0.220	0.830
nuclear-speckles	0.000	0.978	0.013	0.009	0.011	0.005	-	0.231	0.992	0.091	0.600	0.158	0.086	0.743	0.270	0.976	0.455	0.172	0.250	0.143	0.890	0.312	0.989	0.303	0.333	0.317	0.189	0.865	0.338	0.989	0.333	0.355	0.344	0.208	0.865
nucleoli	0.000	0.920	0.039	0.045	0.042	0.021	-	0.505	0.963	0.447	0.613	0.517	0.349	0.888	0.472	0.932	0.700	0.362	0.477	0.313	0.929	0.525	0.960	0.541	0.551	0.546	0.376	0.918	0.564	0.970	0.400	0.829	0.540	0.370	0.902
nucleoli-fibrillar-center	0.000	0.988	0.007	0.006	0.006	0.003	-	0.091	0.844	0.591	0.022	0.042	0.021	0.814	-0.039	0.469	0.273	0.003	0.006	0.003	0.373	-0.058	0.395	0.227	0.002	0.004	0.002	0.352	0.020	0.956	0.091	0.013	0.023	0.012	0.605

SI Table 7: Per-class performance of existing baseline models for level 1 compartments.

	Random						
	MCC	ACC	Recall	Prec	F1	Jacc	AUC
cytoskeleton	0.000	0.872	0.086	0.051	0.064	0.033	-
plasma-membrane	0.000	0.737	0.198	0.107	0.139	0.075	-
cytosol	0.000	0.543	0.368	0.338	0.352	0.214	-
endoplasmic-reticulum	0.000	0.892	0.070	0.044	0.054	0.028	-
golgi-apparatus	0.000	0.907	0.059	0.038	0.046	0.024	-
vesicles	0.000	0.809	0.113	0.101	0.101	0.056	-
mitochondria	0.000	0.830	0.099	0.088	0.093	0.049	-
nucleus	0.000	0.512	0.386	0.448	0.415	0.262	-
nucleoli	0.000	0.908	0.045	0.051	0.048	0.025	-

	MuLocDeep						
	MCC	ACC	Recall	Prec	F1	Jacc	AUC
cytoskeleton	0.359	0.905	0.569	0.286	0.381	0.235	0.877
plasma-membrane	0.574	0.921	0.595	0.642	0.617	0.446	0.904
cytosol	0.408	0.676	0.833	0.513	0.635	0.466	0.785
endoplasmic-reticulum	0.567	0.959	0.663	0.521	0.584	0.412	0.938
golgi-apparatus	0.355	0.964	0.248	0.554	0.343	0.207	0.840
vesicles	0.281	0.835	0.464	0.297	0.362	0.221	0.759
mitochondria	0.764	0.964	0.731	0.839	0.781	0.641	0.950
nucleus	0.538	0.772	0.692	0.776	0.731	0.577	0.860
nucleoli	0.504	0.959	0.423	0.648	0.512	0.344	0.883

	DeepLoc2-ProtT5						
	MCC	ACC	Recall	Prec	F1	Jacc	AUC
cytoskeleton	0.418	0.903	0.697	0.304	0.423	0.268	0.906
plasma-membrane	0.615	0.913	0.767	0.570	0.654	0.486	0.931
cytosol	0.495	0.729	0.860	0.566	0.683	0.518	0.819
endoplasmic-reticulum	0.547	0.948	0.741	0.441	0.553	0.382	0.952
golgi-apparatus	0.310	0.958	0.262	0.418	0.322	0.192	0.871
vesicles	0.286	0.683	0.789	0.212	0.334	0.201	0.811
mitochondria	0.818	0.972	0.779	0.891	0.831	0.711	0.967
nucleus	0.633	0.819	0.736	0.839	0.784	0.645	0.897
nucleoli	0.525	0.959	0.469	0.634	0.540	0.369	0.919

	DeepLoc2-ESM1						
	MCC	ACC	Recall	Prec	F1	Jacc	AUC
cytoskeleton	0.392	0.914	0.590	0.318	0.413	0.260	0.899
plasma-membrane	0.615	0.920	0.720	0.604	0.657	0.489	0.926
cytosol	0.478	0.721	0.849	0.558	0.673	0.507	0.802
endoplasmic-reticulum	0.555	0.953	0.711	0.470	0.566	0.395	0.948
golgi-apparatus	0.408	0.964	0.331	0.545	0.412	0.259	0.885
vesicles	0.301	0.724	0.747	0.231	0.353	0.214	0.808
mitochondria	0.813	0.972	0.752	0.910	0.824	0.700	0.960
nucleus	0.614	0.807	0.680	0.860	0.759	0.612	0.892
nucleoli	0.572	0.964	0.474	0.732	0.576	0.404	0.902

	LAProtT5						
	MCC	ACC	Recall	Prec	F1	Jacc	AUC
cytoskeleton	0.420	0.939	0.487	0.419	0.450	0.291	0.903
plasma-membrane	0.632	0.927	0.703	0.644	0.672	0.506	0.934
cytosol	0.502	0.751	0.803	0.599	0.686	0.522	0.831
endoplasmic-reticulum	0.606	0.970	0.554	0.697	0.617	0.447	0.959
golgi-apparatus	0.334	0.965	0.179	0.667	0.283	0.165	0.885
vesicles	0.299	0.770	0.654	0.252	0.364	0.222	0.822
mitochondria	0.854	0.977	0.818	0.916	0.864	0.761	0.960
nucleus	0.630	0.815	0.689	0.871	0.769	0.625	0.899
nucleoli	0.585	0.967	0.403	0.888	0.554	0.383	0.899

SI Table 8: Per-class performance of existing baseline models for level 2 compartments.

	Random							MuLocDeep							DeepLoc2-ProtT5							DeepLoc2-ESM1							LAProtT5						
	MCC	ACC	Recall	Prec	F1	Jacc	AUC	MCC	ACC	Recall	Prec	F1	Jacc	AUC	MCC	ACC	Recall	Prec	F1	Jacc	AUC	MCC	ACC	Recall	Prec	F1	Jacc	AUC	MCC	ACC	Recall	Prec	F1	Jacc	AUC
cytoskeleton	0.000	0.872	0.086	0.052	0.064	0.033	-	0.350	0.852	0.744	0.220	0.340	0.205	0.881	0.406	0.896	0.703	0.288	0.408	0.257	0.904	0.380	0.895	0.656	0.276	0.389	0.242	0.895	0.449	0.939	0.544	0.426	0.477	0.314	0.916
plasma-membrane	0.000	0.737	0.198	0.107	0.139	0.075	-	0.566	0.918	0.602	0.622	0.612	0.441	0.897	0.622	0.921	0.732	0.606	0.663	0.496	0.932	0.613	0.920	0.710	0.608	0.655	0.487	0.928	0.627	0.932	0.629	0.701	0.663	0.496	0.935
cytosol	0.000	0.543	0.368	0.338	0.352	0.214	-	0.417	0.677	0.848	0.514	0.640	0.471	0.788	0.494	0.739	0.830	0.580	0.683	0.518	0.818	0.481	0.722	0.852	0.559	0.675	0.509	0.801	0.485	0.736	0.817	0.578	0.677	0.512	0.818
endomembrane-system	0.000	0.684	0.217	0.175	0.194	0.107	-	0.540	0.879	0.516	0.716	0.599	0.428	0.848	0.535	0.867	0.607	0.625	0.616	0.445	0.887	0.564	0.882	0.570	0.702	0.629	0.458	0.888	0.558	0.887	0.471	0.802	0.593	0.422	0.902
mitochondria	0.000	0.830	0.099	0.088	0.093	0.049	-	0.749	0.962	0.713	0.830	0.767	0.622	0.959	0.811	0.971	0.773	0.884	0.825	0.702	0.969	0.809	0.971	0.749	0.906	0.820	0.695	0.958	0.854	0.977	0.821	0.914	0.865	0.762	0.965
nucleus	0.000	0.512	0.386	0.448	0.415	0.262	-	0.540	0.773	0.685	0.782	0.730	0.575	0.862	0.636	0.819	0.719	0.855	0.781	0.641	0.895	0.621	0.813	0.726	0.835	0.776	0.635	0.890	0.662	0.832	0.743	0.864	0.799	0.665	0.903
nucleoli	0.000	0.908	0.045	0.051	0.048	0.025	-	0.496	0.954	0.485	0.559	0.519	0.351	0.886	0.504	0.958	0.434	0.634	0.515	0.347	0.916	0.540	0.960	0.474	0.660	0.552	0.381	0.890	0.548	0.965	0.337	0.930	0.494	0.328	0.877

SI Table 9: Per-class performance of existing baseline models for level 3 compartments.

Level	Model	MCC	ACC	Recall	Precision	F1	Jaccard	ROC-AUC
1	Random	0.038	0.734	0.622	0.603	0.625	0.591	-
	MULocDeep	0.483	0.032	0.075	0.287	0.705	0.663	0.028
	DeepLoc2-ProtT5	0.483	0.064	0.522	0.445	0.611	0.581	0.056
	DeepLoc2-ESM1	0.485	0.034	0.623	0.469	0.618	0.599	0.030
	LAProtT5	0.272	0.152	0.256	0.238	0.425	0.411	0.001
2	Random	0.123	0.968	0.942	0.927	0.948	0.934	-
	MULocDeep	0.006	0.764	0.423	0.089	0.335	0.334	0.141
	DeepLoc2-ProtT5	0.088	0.414	0.337	0.208	0.375	0.379	0.139
	DeepLoc2-ESM1	0.031	0.532	0.376	0.116	0.309	0.312	0.187
	LAProtT5	0.041	0.614	0.477	0.013	0.305	0.291	0.136
3	Random	0.116	0.982	0.965	0.931	0.967	0.950	-
	MULocDeep	0.000	0.583	0.131	0.200	0.374	0.348	0.357
	DeepLoc2-ProtT5	0.025	0.668	0.240	0.193	0.381	0.335	0.343
	DeepLoc2-ESM1	0.013	0.651	0.331	0.146	0.354	0.316	0.250
	LAProtT5	0.003	0.724	0.334	0.006	0.301	0.242	0.174

SI Table 10: R^2 values for linear regression of class size in the Combined HPA-UniProt training set against the performance for existing baseline models across various per-class metrics

Model	Level	Agg. Method	Clip Length	Loss	MLP Dropout	Macro AP
ESM2	1	Mean Pool	1024	Sigmoid Focal	0.25	0.422
	2	Light Attention	1024	BCE	0.00	0.651
	3	Light Attention	2048	BCE	0.00	0.717
ESM3	1	Light Attention	1024	Sigmoid Focal	0.00	0.372
	2	Light Attention	1024	BCE	0.00	0.587
	3	Light Attention	2048	BCE	0.25	0.667
ProtBert	1	Mean Pool	2048	BCE	0.25	0.336
	2	Light Attention	2048	BCE	0.00	0.565
	3	Light Attention	1024	BCE	0.00	0.649
ProtT5	1	Multihead Attention	1024	Sigmoid Focal	0.00	0.428
	2	Multihead Attention	2048	Sigmoid Focal	0.00	0.649
	3	Multihead Attention	1024	BCE	0.00	0.709

SI Table 11: Hyperparameters of best performing models in the hyperparameter sweep for all PLM and aggregation strategy combinations. Best model selected by macro AP performance.

	ESM2 - Mean Pool, 1024, Sigmoid Focal							ESM3 - Light Attention, 1024, Sigmoid Focal							ProtBert - Mean Pool, 2048, BCE							ProtT5 - Multihead Attention, 1024, Sigmoid Focal						
	MCC	ACC	Recall	Prec	F1	Jacc	AUC	MCC	ACC	Recall	Prec	F1	Jacc	AUC	MCC	ACC	Recall	Prec	F1	Jacc	AUC	MCC	ACC	Recall	Prec	F1	Jacc	AUC
cytoskeleton	0.507	0.943	0.630	0.455	0.528	0.359	0.918	0.433	0.930	0.583	0.376	0.457	0.296	0.902	0.344	0.891	0.604	0.254	0.358	0.218	0.882	0.444	0.939	0.542	0.418	0.472	0.309	0.909
actin-filaments	0.191	0.975	0.345	0.118	0.175	0.096	0.916	0.340	0.990	0.345	0.345	0.345	0.208	0.916	0.168	0.982	0.241	0.130	0.169	0.092	0.861	0.211	0.972	0.414	0.119	0.185	0.102	0.914
intermediate-filaments	0.00	0.998	0.00	0.00	0.00	0.00	0.771	0.408	0.999	0.167	1.00	0.286	0.167	0.887	0.00	0.998	0.00	0.00	0.00	0.00	0.761	-0.001	0.998	0.00	0.00	0.00	0.00	0.774
centrosome	0.459	0.969	0.416	0.542	0.471	0.308	0.915	0.335	0.953	0.400	0.323	0.357	0.217	0.868	0.238	0.864	0.592	0.137	0.222	0.125	0.867	0.398	0.967	0.352	0.489	0.409	0.257	0.900
microtubules	0.118	0.978	0.154	0.107	0.126	0.067	0.907	0.162	0.969	0.282	0.109	0.157	0.085	0.875	0.107	0.965	0.205	0.073	0.107	0.057	0.877	0.157	0.968	0.282	0.104	0.152	0.082	0.889
plasma-membrane	0.658	0.933	0.720	0.672	0.695	0.533	0.936	0.592	0.921	0.651	0.622	0.636	0.467	0.911	0.568	0.916	0.631	0.599	0.615	0.444	0.910	0.620	0.929	0.639	0.681	0.659	0.491	0.937
cytosol	0.516	0.755	0.823	0.601	0.695	0.532	0.823	0.465	0.738	0.764	0.586	0.663	0.496	0.807	0.380	0.627	0.895	0.473	0.619	0.448	0.780	0.501	0.744	0.828	0.586	0.686	0.522	0.829
endoplasmic-reticulum	0.620	0.968	0.651	0.624	0.637	0.468	0.950	0.567	0.963	0.596	0.576	0.586	0.414	0.941	0.556	0.965	0.536	0.614	0.572	0.401	0.929	0.658	0.972	0.663	0.683	0.673	0.507	0.967
golgi-apparatus	0.475	0.968	0.372	0.643	0.472	0.309	0.906	0.321	0.962	0.228	0.500	0.313	0.185	0.867	0.312	0.965	0.152	0.688	0.249	0.142	0.870	0.413	0.968	0.228	0.786	0.353	0.214	0.905
vesicles	0.378	0.856	0.573	0.361	0.443	0.284	0.812	0.300	0.816	0.550	0.283	0.374	0.230	0.796	0.280	0.727	0.702	0.224	0.340	0.205	0.790	0.384	0.886	0.461	0.435	0.447	0.288	0.841
endosomes	0.263	0.959	0.265	0.304	0.283	0.165	0.886	0.200	0.956	0.205	0.242	0.222	0.125	0.857	0.148	0.934	0.231	0.142	0.176	0.096	0.829	0.297	0.932	0.487	0.221	0.304	0.179	0.893
lysosomes	0.324	0.963	0.311	0.378	0.341	0.206	0.895	0.220	0.908	0.437	0.154	0.228	0.129	0.839	0.265	0.939	0.387	0.224	0.284	0.165	0.863	0.349	0.942	0.521	0.272	0.357	0.218	0.900
peroxisomes	0.639	0.998	0.545	0.750	0.632	0.462	0.976	0.491	0.998	0.364	0.667	0.471	0.308	0.988	0.212	0.997	0.091	0.500	0.154	0.083	0.950	0.403	0.997	0.273	0.600	0.375	0.231	0.991
lipid-droplets	0.455	0.995	0.208	1.00	0.345	0.208	0.947	0.272	0.994	0.125	0.600	0.207	0.115	0.925	0.353	0.994	0.125	1.00	0.222	0.125	0.877	0.490	0.995	0.333	0.727	0.457	0.296	0.970
mitochondria	0.831	0.974	0.779	0.916	0.842	0.727	0.957	0.798	0.970	0.731	0.904	0.809	0.679	0.955	0.745	0.963	0.663	0.881	0.756	0.608	0.951	0.850	0.977	0.812	0.916	0.861	0.756	0.966
nucleoplasm	0.629	0.820	0.727	0.828	0.774	0.632	0.892	0.599	0.804	0.763	0.773	0.768	0.623	0.876	0.521	0.768	0.676	0.752	0.712	0.553	0.854	0.649	0.829	0.797	0.798	0.798	0.664	0.897
nuclear-bodies	0.110	0.649	0.709	0.044	0.083	0.044	0.727	0.051	0.639	0.523	0.033	0.061	0.032	0.615	0.098	0.587	0.744	0.040	0.075	0.039	0.681	0.113	0.716	0.628	0.049	0.091	0.048	0.744
nuclear-membrane	0.456	0.990	0.229	0.917	0.367	0.224	0.850	0.382	0.989	0.167	0.889	0.281	0.163	0.838	0.249	0.988	0.062	1.00	0.118	0.062	0.786	0.499	0.991	0.271	0.929	0.419	0.265	0.880
nuclear-speckles	0.262	0.988	0.242	0.296	0.267	0.154	0.883	0.062	0.990	0.030	0.143	0.050	0.026	0.791	0.217	0.989	0.182	0.273	0.218	0.122	0.863	0.296	0.989	0.273	0.333	0.300	0.176	0.894
nucleoli	0.583	0.971	0.400	0.883	0.551	0.380	0.906	0.469	0.965	0.288	0.803	0.424	0.269	0.872	0.414	0.962	0.259	0.710	0.379	0.234	0.880	0.490	0.966	0.282	0.889	0.429	0.273	0.926
nucleoli-fibrillar-center	0.022	0.720	0.409	0.008	0.017	0.008	0.564	0.027	0.940	0.136	0.014	0.026	0.013	0.530	-0.030	0.357	0.455	0.004	0.008	0.004	0.477	0.035	0.973	0.091	0.023	0.037	0.019	0.608

SI Table 12: Per-class performance of systematic-PLM models models for level 1 compartments.

	ESM2 - Light Attention, 1024, BCE							ESM3 - Light Attention, 1024, BCE						
	MCC	ACC	Recall	Prec	F1	Jacc	AUC	MCC	ACC	Recall	Prec	F1	Jacc	AUC
cytoskeleton	0.437	0.944	0.477	0.456	0.466	0.304	0.923	0.434	0.928	0.590	0.373	0.457	0.296	0.906
plasma-membrane	0.651	0.939	0.607	0.769	0.679	0.514	0.940	0.586	0.925	0.590	0.667	0.626	0.455	0.917
cytosol	0.516	0.767	0.772	0.627	0.692	0.529	0.825	0.460	0.719	0.817	0.558	0.663	0.496	0.810
endoplasmic-reticulum	0.635	0.971	0.620	0.682	0.650	0.481	0.959	0.531	0.962	0.530	0.571	0.550	0.379	0.942
golgi-apparatus	0.373	0.952	0.414	0.382	0.397	0.248	0.897	0.303	0.965	0.145	0.677	0.239	0.135	0.874
vesicles	0.326	0.838	0.534	0.318	0.398	0.249	0.833	0.315	0.853	0.469	0.335	0.390	0.243	0.808
mitochondria	0.844	0.976	0.797	0.921	0.854	0.746	0.963	0.789	0.968	0.725	0.893	0.801	0.668	0.956
nucleus	0.644	0.821	0.690	0.885	0.776	0.634	0.905	0.595	0.799	0.689	0.834	0.755	0.606	0.882
nucleoli	0.630	0.907	0.449	0.917	0.603	0.431	0.918	0.508	0.961	0.383	0.721	0.500	0.333	0.910

	ProtBert - Light Attention, 2048, BCE							ProtT5 - Multihead Attention, 2048, Sigmoid Focal						
	MCC	ACC	Recall	Prec	F1	Jacc	AUC	MCC	ACC	Recall	Prec	F1	Jacc	AUC
cytoskeleton	0.328	0.940	0.323	0.399	0.357	0.217	0.878	0.443	0.943	0.503	0.445	0.472	0.309	0.918
plasma-membrane	0.611	0.928	0.629	0.674	0.651	0.482	0.912	0.619	0.929	0.636	0.682	0.658	0.491	0.938
cytosol	0.432	0.693	0.835	0.529	0.648	0.479	0.782	0.518	0.750	0.847	0.591	0.696	0.534	0.833
endoplasmic-reticulum	0.550	0.966	0.494	0.651	0.562	0.390	0.934	0.675	0.973	0.687	0.691	0.689	0.525	0.963
golgi-apparatus	0.306	0.957	0.269	0.398	0.321	0.191	0.842	0.430	0.969	0.248	0.783	0.377	0.232	0.907
vesicles	0.241	0.585	0.844	0.176	0.291	0.170	0.791	0.398	0.865	0.576	0.385	0.461	0.300	0.834
mitochondria	0.809	0.971	0.749	0.906	0.820	0.695	0.955	0.856	0.978	0.824	0.914	0.867	0.765	0.971
nucleus	0.570	0.788	0.717	0.791	0.752	0.603	0.866	0.652	0.826	0.709	0.879	0.785	0.646	0.905
nucleoli	0.535	0.962	0.423	0.722	0.534	0.364	0.878	0.622	0.969	0.444	0.906	0.596	0.424	0.903

SI Table 13: Per-class performance of systematic-PLM models models for level 2 compartments.

	ESM2 - Light Attention, 1024, BCE							ESM3 - Light Attention, 1024, BCE							ProtBert - Light Attention, 2048, BCE							ProtT5 - Multihead Attention, 2048, Sigmoid Focal						
	MCC	ACC	Recall	Prec	F1	Jacc	AUC	MCC	ACC	Recall	Prec	F1	Jacc	AUC	MCC	ACC	Recall	Prec	F1	Jacc	AUC	MCC	ACC	Recall	Prec	F1	Jacc	AUC
cytoskeleton	0.432	0.939	0.513	0.420	0.462	0.300	0.919	0.426	0.925	0.595	0.360	0.449	0.289	0.907	0.345	0.933	0.400	0.361	0.380	0.234	0.880	0.459	0.948	0.477	0.495	0.486	0.321	0.915
plasma-membrane	0.637	0.935	0.622	0.727	0.670	0.504	0.940	0.597	0.928	0.577	0.699	0.633	0.463	0.915	0.620	0.928	0.654	0.667	0.660	0.493	0.917	0.644	0.932	0.683	0.681	0.682	0.518	0.936
cytosol	0.535	0.765	0.836	0.611	0.706	0.546	0.834	0.476	0.740	0.782	0.587	0.670	0.504	0.815	0.415	0.697	0.787	0.536	0.638	0.468	0.783	0.513	0.763	0.787	0.617	0.692	0.529	0.830
endomembrane-system	0.603	0.888	0.646	0.695	0.669	0.503	0.904	0.577	0.887	0.559	0.735	0.635	0.465	0.875	0.518	0.869	0.540	0.655	0.592	0.420	0.867	0.582	0.890	0.547	0.756	0.635	0.465	0.903
mitochondria	0.845	0.976	0.806	0.912	0.856	0.748	0.966	0.801	0.970	0.755	0.885	0.815	0.688	0.950	0.788	0.968	0.719	0.899	0.799	0.666	0.959	0.856	0.978	0.806	0.934	0.865	0.763	0.963
nucleus	0.641	0.818	0.669	0.899	0.767	0.622	0.906	0.603	0.804	0.722	0.819	0.768	0.623	0.888	0.557	0.781	0.738	0.766	0.751	0.602	0.867	0.660	0.831	0.738	0.865	0.797	0.662	0.905
nucleoli	0.609	0.968	0.449	0.863	0.591	0.419	0.913	0.530	0.961	0.444	0.680	0.537	0.367	0.905	0.545	0.964	0.372	0.839	0.516	0.348	0.874	0.575	0.966	0.367	0.935	0.527	0.358	0.916

SI Table 14: Per-class performance of systematic-PLM models models for level 3 compartments.

Representative Motif	Localization	UniProt ID	Motif Variant	Species	Mean Pairwise Sequence Similarity
QIVSSITA	cytosol	P02550	QIVSSITA	Sus scrofa	97%
		P05214	QIVSSITA	Mus musculus	
		P68369	QIVSSITA	Mus musculus	
		P68363	QIVSSITA	Homo sapiens	
		P81947	QIVSSITA	Bos taurus	
		P81948	QIVSSITA	Bos taurus	
		Q2HJ86	QIVSSITA	Bos taurus	
		Q32KN8	QIVSSITA	Bos taurus	
		Q3ZCJ7	QIVSSITA	Bos taurus	
		Q2XVP4	QIVSSITA	Sus scrofa	
NIFANLFKGLFGKKE	golgi-apparatus	P61204	NIFGNLLKSLIGKKE	Homo sapiens	97%
		P61209	NVFANLFKGLFGKKE	Drosophilia melanogaster	
		P84078	NIFANLFKGLFGKKE	Mus musculus	
		P84079	NIFANLFKGLFGKKE	Rattus norvegicus	
		P84080	NIFANLFKGLFGKKE	Bos taurus	
TVFSFGS	nuclear-membrane	P32499	SVFSFGP	Saccharomyces cerevisiae	37%
		Q10168	PSTFSFGK	Schizosaccharomyces pombe	
		Q7JXF5	TTFSFGT	Drosophilia melanogaster	
		Q7K0D8	SAFSFGF	Drosophilia melanogaster	
		P35658	TVFSFGS	Homo sapiens	
LILLLLL	plasma-membrane	O22920	PVLLLL	Arabidopsis thaliana	32%
		Q75HB1	ALLLLL	Oryza sativa subsp. japonica	
		Q9ESM6	ASILLLL	Mus musculus	
		Q4V9L6	LILLLLL	Homo sapiens	
		Q9NPR2	LLLLLLL	Homo sapiens	
QYSFINQLC	plasma-membrane	Q3KRC6	QYSFINQLC	Rattus norvegicus	64%
		Q498T9	QYSFINQM	Rattus norvegicus	
		Q5U308	YVFINQMC	Rattus norvegicus	
		Q8BGR2	YVFINQMC	Mus musculus	
		Q8R502	QYSFINQM	Mus musculus	
		Q8TDW0	QYSFINQM	Homo sapiens	
		Q6NSJ5	QYSFINQLC	Homo sapiens	
		Q7L1W4	YVFINQMC	Homo sapiens	
RAIAIVSVLVILIS	plasma-membrane	P16389	ARIIAIVSVMVIL	Homo spaiens	79%
		P17659	RGIAIVSVLVILIS	Rattus norvegicus	
		P19024	RAIAIVSVLVILIS	Rattus norvegicus	
		P63142	ARIIAIVSVMVIL	Rattus norvegicus	
		P63141	ARIIAIVSVMVIL	Mus musculus	
		Q61762	RAIAIVSVLVILIS	Mus musculus	
		Q09081	ARIIAIVSVMVIL	Oryctolagus cuniculus	
		P50638	RAIAIVSVLVILIS	Oryctolagus cuniculus	
		Q28293	ARIIAIVSVMVIL	Canis lupus familiaris	
YIINLLVPS	plasma-membrane	A5X5Y0	YVINLLVPS	Homo sapiens	37%
		Q8WXA8	YIINLLVPS	Homo sapiens	
		Q9UGM1	YIVNLLIPC	Homo sapiens	
		P43144	YIVNLLIPC	Rattus norvegicus	
		P54244	YLVNLIIPT	Caenorhabditis elegans	
		O76554	YLVNLIIPT	Caenorhabditis elegans	
ARGYIVT	vesicles	P80560	QGYILT	Mus musculus	59%
		Q60673	EYGYIVT	Mus musculus	
		Q63259	EYGYIVT	Rattus norvegicus	
		Q63475	QGYILT	Rattus norvegicus	
		Q92932	ARGYIVT	Homo sapiens	
DHECCERVVINI	vesicles	P10499	HDDHECCERVVINI	Rattus norvegicus	88%
		P63142	DHECCERVVINI	Rattus norvegicus	
		P16388	HDDHECCERVVINI	Mus musculus	
		P63141	DHECCERVVINI	Mus musculus	
		Q09470	HECCERVVINI	Homo sapiens	

SI Table 15: Fuzzy sequences motifs identified by attention peaks from ProtT5-MHA associated with homologous proteins from three or more species.

**Inference Mode**	**Macro AP**			**Micro AP**			**Macro F1**			**Micro F1**			**Macro Jaccard**			**Micro Jaccard**		
	1	2	3	1	2	3	1	2	3	1	2	3	1	2	3	1	2	3
Train PPI	0.418	0.633	0.694	0.654	0.746	0.781	0.403	0.594	0.654	0.533	0.671	0.712	0.282	0.443	0.504	0.363	0.505	0.553
Test PPI	0.418	0.633	0.695	0.653	0.746	0.781	0.393	0.603	0.659	0.515	0.677	0.717	0.274	0.451	0.509	0.347	0.511	0.558
No PPI	0.432	0.641	0.697	0.665	**0.752**	0.772	**0.406**	0.598	0.656	0.534	0.534	0.713	**0.286**	0.449	0.505	0.364	0.513	0.554
All PPI	0.418	0.633	0.694	0.654	0.746	0.781	0.392	0.598	0.658	0.549	0.676	0.715	0.273	0.447	0.507	0.379	0.510	0.556
ProtT5-MHA	**0.428**	**0.649**	**0.709**	**0.684**	0.748	**0.787**	0.403	**0.622**	**0.669**	**0.585**	**0.690**	**0.718**	0.281	**0.470**	**0.516**	**0.414**	**0.527**	**0.560**

**Inference Mode**	**Accuracy**			**Macro AUC**			**Micro AUC**			**MLRAP**			**Coverage Error**			**Num Labels**		
	1	2	3	1	2	3	1	2	3	1	2	3	1	2	3	1	2	3
Train PPI	0.258	0.594	0.654	0.862	0.899	0.905	0.937	0.933	0.930	0.802	0.834	0.854	2.831	1.942	1.788	1.832	1.384	1.211
Test PPI	0.219	0.603	**0.659**	0.862	0.900	0.905	0.938	0.933	0.930	0.803	0.837	0.854	2.820	1.936	1.788	2.120	1.399	1.205
No PPI	0.248	**0.598**	0.656	0.871	0.905	0.907	0.940	0.935	0.929	0.806	0.833	0.853	2.787	1.930	1.796	2.018	1.324	1.164
All PPI	**0.275**	**0.598**	0.658	0.861	0.899	0.905	0.937	0.932	0.930	0.802	**0.854**	0.854	2.835	1.943	1.787	1.886	1.394	**1.217**
ProtT5-MHA	0.272	0.476	0.537	**0.882**	**0.908**	**0.910**	**0.944**	**0.935**	**0.935**	**0.809**	0.834	**0.861**	**2.726**	**1.930**	**1.743**	**1.852**	**1.314**	1.193

SI Table 16: Averaged metrics for ProtT5-MHA-PPI when doing inference with different subsets of PPI edges compared to the results for ProtT5-MHA. Results are shown for models trained at each level of hierarchical label set. Best performance is bolded.

**Locations**	**TP to FN**	**FN to TP**	**TN to FP**	**FP to TN**
**cytoskeleton**	16	12	52	47
**actin-filaments**	5	2	2	10
**intermediate-filaments**	0	0	1	0
**centrosome**	15	1	16	86
**microtubules**	1	0	28	16
**plasma-membrane**	5	15	40	16
**cytosol**	73	38	139	119
**endoplasmic-reticulum**	0	16	22	1
**golgi-apparatus**	1	8	9	0
**vesicles**	17	29	142	25
**endosomes**	6	1	1	26
**lysosomes**	12	1	10	12
**peroxisomes**	2	1	0	0
**lipid-droplets**	0	0	0	0
**mitochondria**	1	8	8	0
**nucleoplasm**	75	68	100	30
**nuclear-bodies**	8	5	81	251
**nuclear-membrane**	5	1	0	1
**nuclear-speckles**	0	6	13	0
**nucleoli**	10	2	3	7
**nucleoli-fibrillar-center**	1	0	66	551

SI Table 17: Predictions shift per compartment for ProtT5-MHA-PPI without inference to with inference
